# A Bayesian Reformulation of the Extended Drift-Diffusion Model in Perceptual Decision Making

**DOI:** 10.3389/fncom.2017.00029

**Published:** 2017-05-11

**Authors:** Pouyan R. Fard, Hame Park, Andrej Warkentin, Stefan J. Kiebel, Sebastian Bitzer

**Affiliations:** ^1^Department of Psychology, Technische Universität DresdenDresden, Germany; ^2^Bernstein Center for Computational NeuroscienceBerlin, Germany

**Keywords:** perceptual decision making, drift-diffusion model, Bayesian models, parameter fitting, exact input modeling, model comparison, single-trial models

## Abstract

Perceptual decision making can be described as a process of accumulating evidence to a bound which has been formalized within drift-diffusion models (DDMs). Recently, an equivalent Bayesian model has been proposed. In contrast to standard DDMs, this Bayesian model directly links information in the stimulus to the decision process. Here, we extend this Bayesian model further and allow inter-trial variability of two parameters following the extended version of the DDM. We derive parameter distributions for the Bayesian model and show that they lead to predictions that are qualitatively equivalent to those made by the extended drift-diffusion model (eDDM). Further, we demonstrate the usefulness of the extended Bayesian model (eBM) for the analysis of concrete behavioral data. Specifically, using Bayesian model selection, we find evidence that including additional inter-trial parameter variability provides for a better model, when the model is constrained by trial-wise stimulus features. This result is remarkable because it was derived using just 200 trials per condition, which is typically thought to be insufficient for identifying variability parameters in DDMs. In sum, we present a Bayesian analysis, which provides for a novel and promising analysis of perceptual decision making experiments.

## Introduction

Perceptual decision making is a critical cognitive function for everyday life. All the time we have to make decisions about the state of the environment, for example when making a decision whether a traffic light is red. One of the key approaches to understanding perceptual decision making is by explaining the underlying mechanisms involved in the categorization of sensory input (Summerfield et al., 2006). This is usually investigated using two-alternative forced choice tasks, for example in electrophysiological experiments with animals (Shadlen and Newsome, 1996; Hernandez et al., 2002; Roitman and Shadlen, 2002; Romo et al., 2004; Gold and Shadlen, 2007), or in neuroimaging studies with humans (Heekeren et al., 2004, 2008; Gold and Shadlen, 2007; Donner et al., 2009; Siegel et al., 2011; O'Connell et al., 2012; de Lange et al., 2013; Polania et al., 2014). For example, in the widely used random dot motion task participants have to decide about the direction into which visually presented dots move (Newsome and Pare, 1988; Britten et al., 1992; Pilly and Seitz, 2009). Using this and similar tasks, a large body of literature established many important findings about the brain processes involved in perceptual decision making (see Gold and Shadlen, 2007 for review).

On the modeling side, sequential sampling models provide a way to explain the mechanisms of how noisy pieces of information are accumulated until a threshold in evidence is reached (Luce, 1986; Forstmann et al., 2016). This type of models has been proposed to account for behavioral data collected from perceptual decision making tasks in various experimental areas ranging from working memory to aging and clinical applications (Luce, 1986; Smith and Ratcliff, 2004; Gold and Shadlen, 2007; Ratcliff and McKoon, 2008; Voss et al., 2013; Forstmann et al., 2016; Ratcliff et al., 2016). Among such sequential sampling models, the drift-diffusion model (DDM) is probably the most established one that allows modeling the behavioral data of typical perceptual decision making tasks (Ratcliff, 1978; Ratcliff and McKoon, 2008).

The mechanism of how DDM and sequential sampling models accumulate sensory evidence are profoundly connected to Bayesian models of decision making (Dayan and Daw, 2008; Deneve, 2012; Bitzer et al., 2014, 2015; Summerfield and de Lange, 2014). Specifically, a particular formulation of such a Bayesian model, which we here call “BM,” is formally equivalent to a basic version of the DDM, which we call pure DDM “pDDM” (Bitzer et al., 2014). A critical difference between the BM and the pDDM is that the BM explicitly models how observed sensory input is translated into evidence for the decision. We have recently shown that this feature greatly improves fits of response behavior in a perceptual decision making experiment (Park et al., 2016).

In the BM, we showed equivalence only to the pure DDM. But typically experimenters use an extended version, the extended drift-diffusion model (eDDM). The assumption made by using the pDDM is that parameters such as drift rate and bias are constant across trials. The extended DDM goes beyond these assumptions and, in addition, uses parameters such as the inter-trial variability of the drift rate, the bias and the non-decision time (Ratcliff, 1981; Ratcliff and Rouder, 1998; Ratcliff and Tuerlinckx, 2002; Ratcliff and McKoon, 2008; Voss et al., 2013; Forstmann et al., 2016). These inter-trial variabilities have been reported to allow for different patterns of response time (RT) distributions for both correct and error responses and for a better fit to experimental data (Ratcliff and Rouder, 1998; Ratcliff and Tuerlinckx, 2002; Ratcliff and McKoon, 2008; Voss et al., 2013; Forstmann et al., 2016).

Here, we propose an extended Bayesian model (eBM) that incorporates the inter-trial variability assumptions made in the eDDM. The usefulness of the eBM is that it combines the variability parameters of the eDDM with the additional feature of being able to model the translation of sensory input to evidence for a decision (Park et al., 2016). To do this, we use the previously established BM as a basis to derive the more complex eBM. To validate the eBM, we show, using simulations, that the eBM, just as the eDDM, can model two experimentally well-established behavioral effects: (i) the effect of task difficulty on RT and accuracy (Philiastides et al., 2006; Ratcliff and McKoon, 2008; Forstmann et al., 2016) and (ii) differences in RTs between correct and error responses (Luce, 1986; Ratcliff and Rouder, 1998; Ratcliff and McKoon, 2008; Forstmann et al., 2016). In addition, we demonstrate fitting of the eBM to concrete multi-subject behavioral data (Park et al., 2016). There are two main findings. Firstly, we find that constraining the eBM with evidence extracted from the stimulus provides for a better model, as already shown for the BM (Park et al., 2016). Secondly, using this exact input version of the eBM, we show that, with just 200 trials per condition, the additional variability parameters as used in the extended DDM provide for a better model for the behavioral data of the easy conditions.

## Models

In the following, we will describe the particular models considered here in more detail; starting with variants of the DDM, followed by their Bayesian versions and a description of how one can easily change the sensory input in these Bayesian models to improve model fits to behavioral data.

### Pure drift-diffusion model (pDDM)

We define the pDDM (Bogacz et al., 2006; Wagenmakers et al., 2007) as a discretized version of a simple Wiener diffusion process:

(1) $$\begin{array}{l} y_{t}^{d}-y_{t-△t}^{d}=v^{d}△t+\sqrt{△t}s^{d}\varepsilon_{t}^{d} \end{array}$$

where $y_{t}^{d}$ is the decision variable at the given time *t*, △*t* is the time-step length, *v*^*d*^ stands for the drift rate, *s*^*d*^ is the diffusion rate, and $\varepsilon_{t}^{d}\sim N\left(0,1\right)$ is a standard normally distributed noise variable.

Applied to two-alternative perceptual decision making tasks, the model describes the decision process as a random walk in which steps have mean *v*^*d*^△*t* and variance △*ts*^*d*^2. The walk continues until it crosses one of the bounds at ±*B*^*d*^ which determines the choice and response time. For a non-biased decision the starting point is set mid-way between the two boundaries at 0. However, the model can also account for the preference of participants toward one of the alternatives using a bias parameter, *z*^*d*^, which shifts the starting point toward one of the two boundaries. The values of *z*^*d*^ should be constrained between the upper and lower boundary values. The value of the drift rate models the difficulty of the decision: Decreasing the drift rate increases the number of errors and slows down decisions, that is, small drift rates model hard decisions (Ratcliff and McKoon, 2008; Voss et al., 2013). Furthermore, the pDDM allows for an additional delay which may result from basic sensory processing and motor production. This delay is modeled as a non-decision time component, $T_{nd}^{d}.$ An overview of pDDM parameters is given in Table 1.

**Table 1:** **Model parameters for the extended Bayesian model (eBM) and the extended drift-diffusion model (eDDM)**.

**eBM/BM**			**eDDM/pDDM**		
**Parameter**	**Name**	**Constraint**	**Parameter**	**Name**	**Constraint**
λ^*b*^	Bound	–	*B*^*d*^	Boundary	–
${\overset{-}{\sigma }}^{b}$	Mean noise level	–	${\overset{-}{v}}^{d}$	Mean drift rate	–
$\eta_{N}^{b}†$	Noise level variability	–	^**η**^^*d*^†	Drift variability	–
${\overset{-}{p}}_{0}^{b}$	Mean prior	${\overset{-}{p}}_{0}^{b}<\lambda^{b}$	${\overset{-}{z}}^{d}$	Mean bias	$-B^{d}<{\overset{-}{z}}^{d}<B^{d}$
$s_{P}^{b}†$	Prior variability	$s_{P}^{b}<2\left(\lambda^{b}-{\overset{-}{p}}_{0}^{b}\right)$	$s_{z}^{d}†$	Bias variability	$s_{z}^{d}<2\left(B^{d}\pm Z^{d}\right)$
$T_{nd}^{b}$	Mean non-decision time	–	$T_{nd}^{d}$	Mean non-decision time	–
$s_{t}^{b}$	Non-decision time variability	–	$s_{t}^{d}†$	Non-decision time variability	–
${\hat{\sigma }}^{b}$	Internal uncertainty	–	–	–	–
$\pi_{l}^{b}$	Lapse probability	–	–	–	–
$\pi_{to}^{b}$	Time-out lapse probability	–	–	–	–

*Each row lists those two parameters which are related between the two models. The parameters indicated by † are additional to the pure models, i.e., the pure drift-diffusion model (pDDM) and the Bayesian model (BM; see Park et al., 2016). For the exact mathematical relationships, see Equations (2–19)*.

### Extended drift-diffusion model (eDDM)

The eDDM has mainly been developed by Ratcliff starting in the 1970s (see Ratcliff et al., 2016 for review). The currently most often used version adds the following inter-trial parameter variabilities (see Table 1) to a continuous version of the pDDM:

- The drift rate is drawn from a Gaussian distribution $v^{d}\sim N\left({\bar{v}}^{d},\eta^{d}\right)$ where ${\bar{v}}^{d}$ is the mean drift rate, and η^*d*^ is the variability of the drift rate.
- The bias is drawn from a uniform distributions $z^{d}\sim U\left({\bar{z}}^{d}-s_{z}^{d}/2,{\bar{z}}^{d}+s_{z}^{d}/2\right)$ where $s_{z}^{d}$ is the variability of bias. Values of $s_{z}^{d}$ also should be constrained so that the samples from this uniform distribution does not exceed any of the boundaries, ±*B*^*d*^.
- The non-decision time is drawn from a uniform distribution $T_{nd}^{d}\sim U\left({\bar{T}}_{nd}^{d}-s_{t}^{d}/2,{\bar{T}}_{nd}^{d}+s_{t}^{d}/2\right)$ where $s_{t}^{d}$ is variability of non-decision time.

Variability of the drift rate can model response distributions in which error responses are slower than correct responses while variability in bias can explain faster errors (Ratcliff and Rouder, 1998; Ratcliff and McKoon, 2008). Variability of non-decision time has been shown to improve model fits in the presence of contaminant RTs (Ratcliff and Tuerlinckx, 2002).

### Bayesian model (BM)

We have previously introduced the BM as a Bayesian decision model which is equivalent to the pure DDM (Bitzer et al., 2014). The BM describes the decision making process as probabilistic inference about the identity of a stimulus based on the decision maker's assumptions about the possible identities. The BM captures these assumptions in probabilistic generative models which define how likely particular sensory observations are for each considered alternative. Consequently, one of the major advantages of this model is that it establishes a direct link between sensory observations and the decision process. As we have shown before, modeling this link provides for greatly improved models and provide for better fits to the behavioral data (see Park et al., 2016).

**Figure 1:**
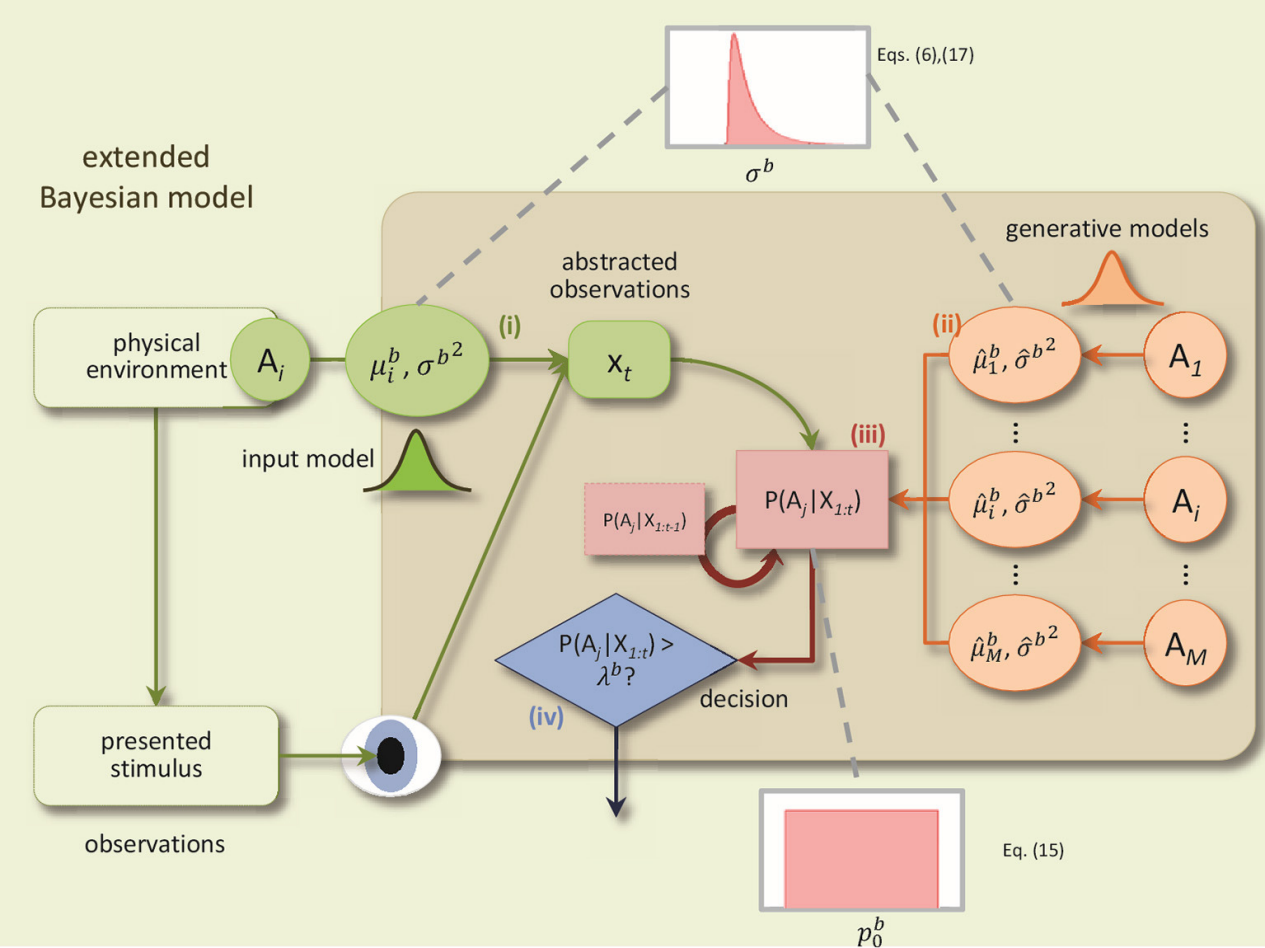
**A schematic of the BM and its extensions**. The BM consists of: (i) an input process which translates the sensory stimulus into an abstract representation, *x*_*t*_, which is drawn from a Gaussian distribution with the mean $\mu_{\text{i}}^{\text{b}}$ and the variance Δ*tσ^b^*^2^, (ii) the generative models are used to compute the likelihood of the current observation under different alternatives, *P*(*x*_*t*_|*A*_*i*_), (iii) Bayesian inference computes recursively the posterior beliefs *P*(*A*_*i*_|*X*_1:*t*_) given the previous beliefs, (iv) decisions are made using the policy which compares, at each time-step, for each alternative, the posterior belief with the bound. In addition, to derive the extended BM, two specific extensions are added to incorporate the variabilities of the noise level and the prior: The noise level value for each trial is randomly distributed according to an inverse Gaussian distribution. Consequently, the internal uncertainty value for each trial is computed using the corresponding noise level value and Equation (6). The prior parameter for each trial is drawn from a uniform distribution (Adapted and modified with permission from Bitzer et al., 2014).

The BM consists of four components: (1) a noisy sensory input process, (2) probabilistic generative models for each alternative, (3) an evidence accumulation process based on Bayesian inference, and (4) a decision policy; see Figure 1 for a schematic.

The sensory input process models the noisy stimulus features that the brain observes. In particular, feature values are sampled at each time point *t* within a trial from a Gaussian distribution with mean $\mu_{t}^{b}$ and variance Δ*t*σ^*b*^2 where Δ*t* is the time-step length, as before. For example, a basic, DDM-equivalent model of a stimulus which ignores stimulus changes over time, would set $\mu_{t}^{b}$ to a fixed value throughout the trial. This value is associated with the stimulus identity in that trial. The noise standard deviation σ^*b*^ (also referred to as “noise level” in the following) captures the amount of noise or unknown variability in the observed stimulus features that the brain observes. The noise level, thus, captures variability in the stimulus as well as unspecific “neural noise.”

The generative models within the BM mirror the input process. They, too, are Gaussian with mean ${\hat{\mu }}_{i}^{b}$ and variance $\Delta t{\hat{\sigma }}^{b^{2}}$ and capture the decision maker's assumption that observed stimulus features will be sampled from the Gaussian corresponding to alternative *i* when the stimulus has identity *i*. We also interpret the standard deviation ${\hat{\sigma }}^{b}$ as the internal uncertainty of the decision maker about the association of stimulus feature observations with decision alternatives. Note that in the case of two-alternative forced choice tasks there are only two alternatives, but the model itself is not limited to this number.

Given prior beliefs of the decision maker about the stimulus identity, *p*(*A*_*i*_), and a sequence of observed stimulus features, *X*_1:*t*_, the BM uses Bayesian inference to compute the corresponding posterior beliefs *p*(*A*_*i*_|*X*_1:*t*_) at each time point *t* (see Bitzer et al., 2014 for details). For just two alternatives the prior beliefs can be represented by a single value $p_{0}^{b}=p\left(A_{1}\right)=1-p\left(A_{2}\right)$ which is the prior probability of the first alternative.

The decision policy in the BM is a criterion which makes a decision when any of the posterior beliefs *p*(*A*_*i*_|*X*_1:*t*_) first reaches the decision bound λ^*b*^. The alternative *i* for which this is the case will be the selected choice. Finally, a non-decision time component, $T_{nd}^{b}$, is added to the time point identified by the decision criterion to compute the RT.

To summarize, the principle parameters of the BM are the noise level σ^*b*^, the internal uncertainty ${\hat{\sigma }}^{b}$, the prior belief $p_{0}^{b}$, the decision bound λ^*b*^ and the non-decision time $T_{nd}^{b}$ (see Table 1 for overview of parameters). The means $\mu_{i}^{b}$ and ${\hat{\mu }}_{i}^{b}$ will either be determined from the stimulus used in an experiment, or are set to 1 and -1 for the different alternatives. By choosing these values we followed the original proposal in Bitzer et al. (2014). However, this choice is arbitrary and we could have chosen other values without changing results provided that we scale the noise level and bound appropriately (cf. Section Internal Uncertainty and Arbitrary Scaling of Model Parameters). Additionally, we have previously introduced three extra parameters: variability of non-decision time, $s_{t}^{b}$, lapse probability, $\pi_{l}^{b}$, for modeling entirely random lapses, and time out lapse probability, $\pi_{to}^{b}$ (Park et al., 2016; see Section Methods for more details).

### Extended bayesian model (eBM)

We will now derive the eBM from the BM by incorporating the inter-trial variability assumptions for parameters similar to the eDDM.

As the eDDM introduced inter-trial parameter variability to the pDDM, we must now identify equivalent inter-trial variabilities of BM parameters. As we will see, this is not straightforward and requires approximations. First, we map the eDDM parameter distributions through the equivalence equations of Bitzer et al. (2014). These equations are:

(2) $${\hat{\sigma }}^{b^{2}}=\left|\mu_{i(r)}^{b}\;\frac{{\hat{\mu }}_{1}^{b}-{\hat{\mu }}_{2}^{b}}{{\left(\Delta \;t\right)}^{2}v^{d}}\right|$$

(3) $$\begin{array}{l} \sigma^{b}=\;|\frac{s^{d}△t}{{\hat{\mu }}_{1}^{b}-{\hat{\mu }}_{2}^{b}}|{\hat{\sigma }}^{b^{2}} \end{array}$$

(4) $$\begin{array}{l} \lambda^{b}=\;\frac{e^{B^{d}}}{1+e^{B^{d}}} \end{array}$$

(5) $$\begin{array}{l} p_{0}^{b}=\;\frac{e^{z^{d}}}{1+e^{z^{d}}} \end{array}$$

$\mu_{i\left(r\right)}^{b}$ is the mean feature value in the *r*-th trial, i.e., *i*(*r*) is a function which returns the identity of the alternative from which stimulus features were generated in trial *r*. Throughout this paper, we assume that for each alternative *i*, we have ${\hat{\mu }}_{i}^{b}=\mu_{i}^{b}.$

These equations state that the BM prior $p_{0}^{b}$ is related to the pDDM bias *z*^*d*^ and that both noise level σ^*b*^ and internal uncertainty ${\hat{\sigma }}^{b}$ depend on drift *v*^*d*^. Specifically, Equation (3) states that the internal uncertainty is coupled to the noise level when translating between pDDM and BM parameters:

(6) $$\begin{array}{l} {\hat{\sigma }}^{b^{2}}=\frac{\sigma^{b}}{△t\;s^{d}}\;|{\hat{\mu }}_{1}^{b}-{\hat{\mu }}_{2}^{b}| \end{array}$$

In the following, we will derive the eBM equations for the three required variabilities of the prior, of the noise level and the non-decision time. The variability of non-decision time in the eBM can be directly determined from the eDDM, because both eDDM and eBM share the same non-decision time parameters.

#### Translating the eDDM bias into the eBM prior

In the eDDM, the inter-trial variability of the bias is represented by a random variable, *Z*^*d*^, with uniform distribution with mean ${\bar{z}}^{d}$ and spread $s_{z}^{d}$:

(7) $$f_{Z^{d}}\left(z^{d}\right)=\{\begin{array}{c} 1/s_{z}^{d}\\ 0 \end{array}\begin{array}{c} \left({\bar{z}}^{d}-s_{z}^{d}/2\right)\leq \;z^{d}\leq \left({\bar{z}}^{d}+s_{z}^{d}/2\right)\\ \;otherwise \end{array}$$

We consider the random variable *Z*^*d*^, only in the range $\left({\bar{z}}^{d}-s_{z}^{d}/2\right)\leq Z^{d}\leq \left({\bar{z}}^{d}+s_{z}^{d}/2\right).$ We define the random variable $P_{0}^{b}$ for the prior probability as the equivalent parameter to bias in eBM:

(8) $$\begin{array}{l} P_{0}^{b}=u_{1}\left(Z^{d}\right)=\;\frac{e^{Z^{d}}}{1+e^{Z^{d}}} \end{array}$$

Note that *Z*^*d*^ and $P_{0}^{b}$ stand for random variables while *z*^*d*^ and $p_{0}^{b}$ represent their respective values. Since *u*_1_ is an invertible function, we can write:

(9) $$\begin{array}{l} {{\text{Z}}^{d}\;=u}_{1}^{-1}\left(P_{0}^{b}\right)=\log\frac{P_{0}^{b}}{1-P_{0}^{b}} \end{array}$$

By applying a change of variables, we obtain the exact probability density function representing the variability of prior $p_{0}^{b}$ in the eBM corresponding to the variability of bias *z*^*d*^ in the eDDM:

(10) $$\begin{array}{l} f_{P_{0}^{b}}\left(p_{0}^{b}\right)=f_{Z^{d}}\left(u_{1}^{-1}\left(p_{0}^{b}\right)\right).\left|\frac{du_{1}^{-1}\left(p_{0}^{b}\right)}{dp_{0}^{b}}\right|\\ \;=\left(\frac{1}{s_{z}^{d}\;.\;(p_{0}^{b}\text{ -}{p_{0}^{b}}^{2})}\right) \end{array}$$

where $u_{1}\left({\bar{z}}^{d}\;-\;s_{z}^{d}/2\right)\leq p_{0}^{b}\leq u_{1}\left({\bar{z}}^{d}\;+\;s_{z}^{d}/2\right).$

#### Translating the eDDM drift rate into the eBM noise level

Here, we will derive how drift variability relates to variability of the noise level. Note that as the noise level and internal uncertainty are dependent on each other, we could have equally derived how drift variability relates to variability of internal uncertainty without changing the resulting predictions for choice and RT.

In the eDDM, the inter-trial variability of the drift rate is represented by a Gaussian distribution with mean ${\bar{\nu }}^{d}>0$ and standard deviation η^*d*^> 0:

(11) $$\begin{array}{l} f_{\text{V}}\left(\nu^{d}\right)=\frac{1}{\sqrt{2\pi }\eta^{d}}\exp\left(\frac{-{\left({\text{ν}}^{d}-{\overset{-}{\text{ν}}}^{d}\right)}^{2}}{2\eta^{d^{2}}}\right) \end{array}$$

We, then, define the random variable Σ^*b*^ for the noise level as the equivalent parameters to the drift rate in the eBM. By combining Equations (2, 3) and assuming ${\hat{\mu }}_{1}^{b}>{\hat{\mu }}_{2}^{b}$, we have:

(12) $$\begin{array}{l} \Sigma^{b}=u_{2}\left(V^{d}\right)=\;\left|\frac{\mu_{i(r)}^{b}}{V^{d}}\right|\frac{\left({\hat{\mu }}_{1}^{b}-{\hat{\mu }}_{2}^{b}\right)}{{\left(\Delta t\right)}^{2}}.\;\frac{s^{d}\Delta t}{\left({\hat{\mu }}_{1}^{b}-{\hat{\mu }}_{2}^{b}\right)}\\ \;=\;\left|\frac{\mu_{i(r)}^{b}}{V^{d}}\right|.\;\frac{s^{d}}{\Delta t}=\frac{s^{d}}{\Delta t\;V^{d}}\;.\;\mu_{i(r)}^{b}=\frac{c}{V^{d}} \end{array}$$

where $c=s^{d}\mu_{i\left(r\right)}^{b}/△t$ and $\mu_{i\left(r\right)}^{b}/v^{d}$ is always positive, because their signs both signal the identity of the stimulus associated with the current sensory input. Again, *V*^*d*^ and Σ^*b*^ are random variables with *v*^*d*^ and σ^*b*^^2^ as their values, respectively. Inverting $u_{2}\left(V^{d}\right)$ we get:

(13) $$\begin{array}{l} {V^{d}=u}_{2}^{-1}\left(\Sigma^{b}\right)=\;\frac{s^{d}}{△t\Sigma^{b}}.\;\mu_{i\left(r\right)}^{b}=\frac{c}{\Sigma^{b}} \end{array}$$

We can again apply a change of variables to determine the exact probability density function representing the variability of the noise level σ^*b*^ in the eBM corresponding to the variability of drift ν^*d*^ in the eDDM:

(14) $$\begin{array}{l} f_{\Sigma^{b}}\left(\sigma^{b}\right)=f_{V^{d}}\left(u_{2}^{-1}\left(\sigma^{b}\right)\right)|\frac{du_{2}^{-1}\left(\sigma^{b}\right)}{d\sigma^{b}}|=f_{V}\left(\frac{c}{\sigma^{b}}\right)\frac{\left|c\right|}{\sigma^{b^{2}}}\\ =\left(\frac{1}{\sqrt{2\pi }\;\eta^{d}}\text{exp}\left(-{\left(\frac{c}{\sigma^{b}}-{\bar{\nu }}^{d}\right)}^{2}/2{\eta^{d}}^{2}\right)\right).\left(\frac{\left|c\right|}{\sigma^{b^{2}}}\right) \end{array}$$

where *f*_*V*_ is the Gaussian density of Equation (11). Note that *c* becomes negative for samples in which the drift is negative due to the connection to the sensory input through $\mu_{i\left(r\right)}^{b}$.

#### Approximating eBM parameters

Equations (10, 14) depend non-trivially on parameters of the eDDM, such as the drift variability η^*d*^. However, our aim is to formulate variability of eBM parameters without reference to the corresponding eDDM parameters and use standard probability densities only to represent the parameter variabilities. In this way, we can provide for variability parameters that intuitively quantify the inter-trial variability of the corresponding parameters in the Bayesian model. Therefore, we propose simple approximations of the exact probability densities in Equations (10, 14). The resulting eBM will have the same number of variability parameters as the eDDM and the parameters will have the same qualitative interpretation.

##### Variability of prior

**Figure 2:**
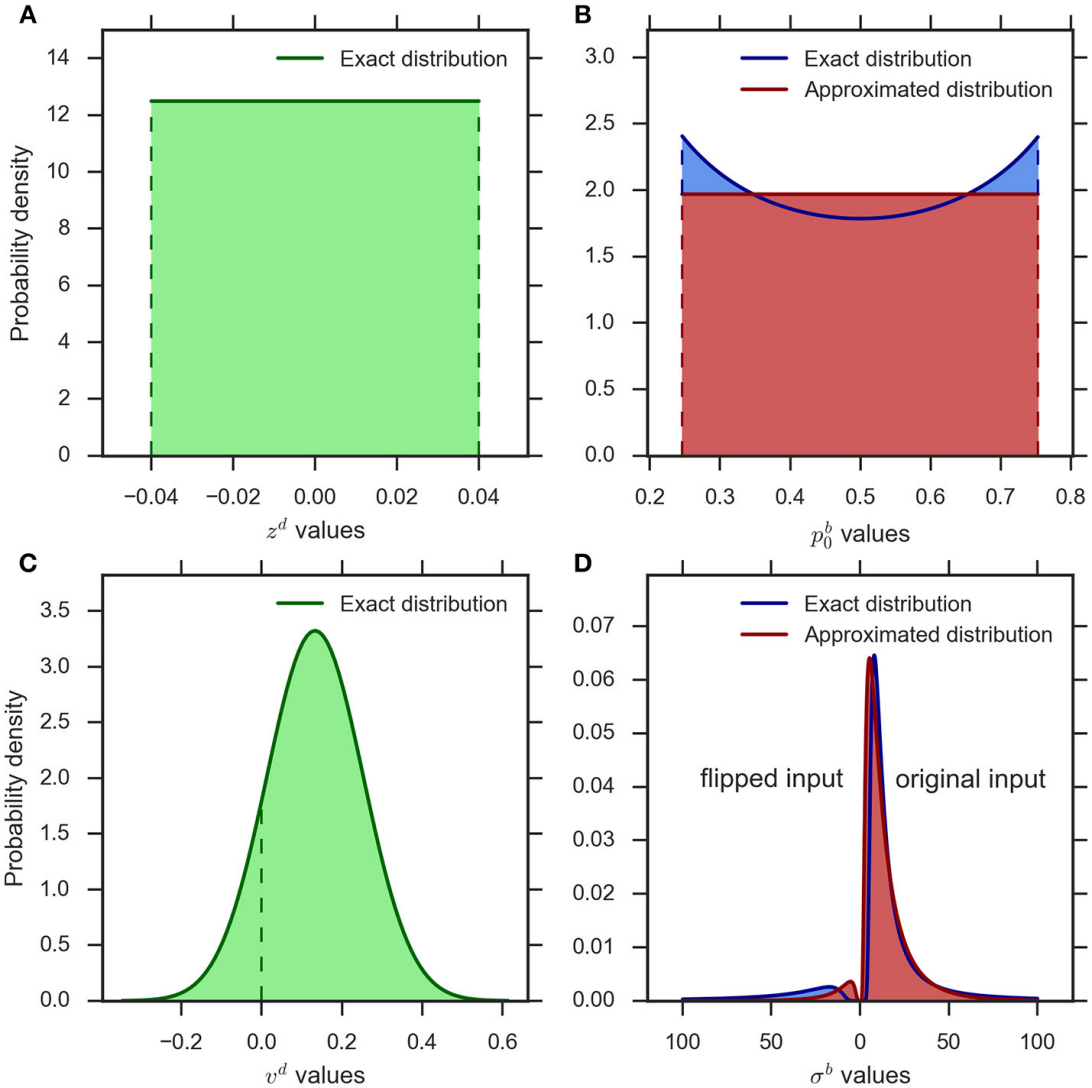
**An example illustration of the distributions implementing parameter variability in the eDDM and eBM. (A)** uniform distribution for the bias parameter in the eDDM (${\overset{-}{z}}^{d}=0,s_{z}^{d}=0.08$) which is translated into **(B)** the exact distribution for the prior parameter in the eBM (blue curve). We approximate this distribution with a uniform distribution (red curve) (${\overset{-}{p}}_{0}^{b}=0.5,s_{P}^{b}=0.508$). **(C)** The Gaussian distribution for the drift rate parameter in the eDDM (${\overset{-}{\nu }}^{d}=0.133,\eta^{d}=0.12$) which is translated into **(D)** the exact distribution over the noise level in the eBM (blue curve). We approximate this distribution with an inverse Gaussian distribution (red curve) (${\overset{-}{\sigma }}^{b}=16.5,\eta_{N}^{b}=0.023$). The values on the left-hand side of 0 represent the noise values for the “flipped input” whereas the noise values on the right-hand side of 0 stand for “original input.”

Figure 2A shows a typical example of the distribution of the bias parameter in the eDDM (cf. Wagenmakers et al., 2007). The corresponding exact distribution of the prior in the eBM is shown in blue in Figure 2B. The exact distribution assigns more probability mass to values close to 0 or 1 than to the center values around 0.5. As the spread between these minimum and maximum density values is moderate in relation to its average within typical ranges of $p_{0}^{b}$, we chose to approximate the density with a simple uniform density (cf. Figure 2B, red):

(15) $$f_{P_{0}^{b}}\left(p_{0}^{b}\right)=\{\begin{array}{c} 1/s_{P}^{b}\;\\ 0 \end{array}\begin{array}{c} {\bar{p}}_{0}^{b}-s_{P}^{b}/2\leq p_{0}^{b}\leq {\bar{p}}_{0}^{b}+s_{P}^{b}/2\\ \;otherwise \end{array}$$

where $s_{P}^{b}$ denotes the spread of the uniform distribution and ${\bar{p}}_{0}^{b}$ represents its mean. Note that values of $s_{P}^{b}$ should be constrained in a way that the sampled prior does not exceed the model bound, λ^*b*^ (see Table 1).

##### Variability of noise level

Figure 2C shows an example distribution of the eDDM drift rate, ν^*d*^, given typical values for ${\bar{\nu }}^{d}$ and η^*d*^ (cf.Wagenmakers et al., 2007). As shown here, the variability of drift may also include values with a different sign than the sign of the mean ${\overset{-}{\nu }}^{d}.$ In Figure 2D we visualize the corresponding exact distribution (blue) of the noise level given in Equation (14), but separate the parts of the distribution that correspond to positive and negative drift rates by plotting them on the right- and left-hand sides of 0, respectively. In the Bayesian model negative drift rates correspond to sampling input from the alternative that does not represent the stimulus in that trial. To approximate the density of Equation (14) we, therefore, need to find a noise level variability parameter with the following properties: (i) for large values the range of sampled noise levels is large, (ii) the resulting density is only defined over positive values, (iii) the resulting density has heavy tails when the variability value is large in relation to the mean (cf. Figure 2D), (iv) the resulting density becomes more Gaussian-like when the variability value is small in relation to the mean, and (v) each variability value determines a probability with which the mean of the input is flipped to the other alternative and this probability should approximate the corresponding probability in the exact distribution.

Because the inverse Gaussian distribution fulfills points (i–iv), we have based our approximation of Equation (14) on it:

(16) $$\begin{array}{l} f_{\Sigma^{b}}\left(\sigma^{b}\right)=IG\left({\overset{-}{\sigma }}^{b},\zeta_{N}^{b}\right)=\sqrt{\frac{\zeta_{N}^{b}}{2\pi \sigma^{b^{3}}}}.\exp\left(\frac{-\zeta_{N}^{b}{\left(\sigma^{b}-{\overset{-}{\sigma }}^{b}\right)}^{2}}{2{\overset{-}{\sigma }}^{b^{2}}\sigma^{b}}\right) \end{array}$$

where ${\overset{-}{\sigma }}^{b}>0$ is the mean and $\zeta_{N}^{b}>$ 0 is the shape parameter of the inverse Gaussian distribution. See Figure 2D (red) for an inverse Gaussian approximation of the corresponding exact distribution.

The shape parameter of the inverse Gaussian distribution, $\zeta_{N}^{b}$, relates to the precision of the distribution. As we are interested in the variability of the noise level, we define $\eta_{N}^{b}=1/\zeta_{N}^{b}$ as the noise level variability parameter in the eBM. Consequently, we re-write Equation (16) as a function of the variability of noise level:

(17) $$\begin{array}{l} f_{\Sigma^{b}}\left(\sigma^{b}\right)=IG\left({\bar{\sigma }}^{b},1/\eta_{N}^{b}\right)\\ \;=\sqrt{\frac{1}{2\pi \eta_{N}^{b}\sigma^{b^{3}}}}.\exp\left(\frac{-{\left(\sigma^{b}-{\bar{\sigma }}^{b}\right)}^{2}}{2{\bar{\sigma }}^{b^{2}}\sigma^{b}\;\eta_{N}^{b}}\right) \end{array}$$

To complete the approximation (point v) we need to approximate the probability of negative drift rates for a given mean drift, ${\overset{-}{\nu }}^{d}$, and drift variability, η^*d*^. As the distribution of drift rates is Gaussian, this probability is simply given by the Gaussian cumulative distribution function:

(18) $$\begin{array}{l} PNT^{d}=\text{Φ}\left(v^{d}=0\right)=\frac{1}{2}\left(1+\text{erf}\left(\frac{-{\overset{-}{\nu }}^{d}}{\eta^{d}\sqrt{2}}\right)\right) \end{array}$$

where erf is the error function. Equation (18) depends on the parameters only via the ratio ${\overset{-}{\nu }}^{d}/\eta^{d}$, i.e., the ratio of mean to standard deviation of the distribution. The mean of the inverse Gaussian distribution defined in Equation (17) is ${\overset{-}{\sigma }}^{b}$ while its variance is ${\overset{-}{\sigma }}^{b^{3}}\eta_{N}^{b}$. The ratio of mean to standard deviation, thus, is equal to $1/\sqrt{{\overset{-}{\sigma }}^{b}\eta_{N}^{b}}$ leading to the following approximation of the probability of a flipped input given mean noise level ${\overset{-}{\sigma }}^{b}$and noise level variability $\eta_{N}^{b}$:

(19) $$\begin{array}{l} PNT^{b}\approx \frac{1}{2}\left(1+\text{erf}\left(-\sqrt{\frac{1}{2\eta_{N}^{b}{\overset{-}{\sigma }}^{b}}}\right)\right) \end{array}$$

Supplementary Figure 1 shows that this approximation behaves qualitatively very similar when the corresponding parameters are manipulated within the considered ranges.

When predicting a decision with the model, the computed proportion represents the probability with which the sign of input features $\mu_{i\left(r\right)}^{b}$ for the given trial is flipped in the eBM. Figure 2D (red) conceptually illustrates the complete approximation resulting from the application of this procedure to samples from the inverse Gaussian distribution, Equation (17), in comparison with the exact distribution derived from the eDDM (blue), Equation (14).

#### Internal uncertainty and arbitrary scaling of model parameters

Equation (6) states that we should determine the value of the internal uncertainty ${\hat{\sigma }}^{b}$ from the value of the noise level, when incorporating the variability of drift in the eBM. However, the equation also depends on the eDDM diffusion parameter *s*^*d*^. Typically, this parameter is chosen to be constant in applications of the eDDM and is arbitrarily set to *s*^*d*^ = 0.1 (Ratcliff and McKoon, 2008). We have argued previously that this setting is unintuitive in the context of the BM (Bitzer et al., 2014) and use here *s*^*d*^ = 2.8 which leads to more intuitive bound values of λ^*b*^ ≈ 0.8 instead of very small values of λ^*b*^ ≈ 0.52.

Coupling the internal uncertainty ${\hat{\sigma }}^{b}$ to the noise level σ^*b*^ through Equation (6) deviates from our previous approaches of letting ${\hat{\sigma }}^{b}$ vary freely (Bitzer et al., 2014), or fixing it to a constant, stimulus-derived value (Park et al., 2016). Coupling ${\hat{\sigma }}^{b}$ to σ^*b*^ is necessary in the eBM to implement the effect of slow errors, that is, the effect that errors are slower than correct responses (cf. **Figure 6**). This is necessary, because varying the noise level alone induces more errors and makes responses overall faster or reduces errors while making responses overall slower, but it is not possible to simultaneously reduce errors and make responses faster by changing the noise level. This latter effect, however, is needed to induce slow errors: Allowing inter-trial variability of drift in the eDDM mixes response distributions with many errors and slow responses (small drift) with response distributions with few errors and fast responses (large drift; Ratcliff and McKoon, 2008). This mixing results in a response distribution with slow errors. By coupling internal uncertainty to noise level in the eBM, as in Equation (6), we achieve the same effect.

### Exact input models

One advantage of translating various versions of the DDM to a Bayesian formulation is that the Bayesian model describes how sensory input features are translated to evidence. This means that one can model the trial-wise stimulus and improve model fits profoundly (Park et al., 2016).

Specifically, the Bayesian model defines a sensory input process (green parts in Figure 1) together with a mechanism which prescribes how this sensory input needs to be interpreted with respect to the decision (through the generative models, orange parts in Figure 1). We can directly link the sensory input process in the model to the stimulus presented in any given trial by equating the sensory input at a particular time point within the trial with a feature value extracted from the stimulus. For example, in the study of Park et al. (2016) the stimulus consisted of a single white dot which jumped around one of two targets (see Park et al., 2016; Figure 1). Consequently we used the changing dot location as mean input feature values $\mu_{i}^{b}$ and set the properties of the internal generative models to the corresponding values of the distributions which generated the stimulus. We called the resulting model the exact input model (EXaM).

We here use the same modeling strategy in the extended exact input model (eEXaM). The resulting eEXaM is exactly the same as the eBM except for the mechanism of representing the sensory stimuli: The eBM uses only average information about the stimulus in a trial, as the standard eDDM, whereas the eEXaM uses the particular sequence of stimulus features (dot positions) shown in single trials to predict responses. Therefore, the eEXaM combines both the inter-trial variability in parameters and the within-trial variability of stimulus feature values.

### Summary

**Figure 3:**
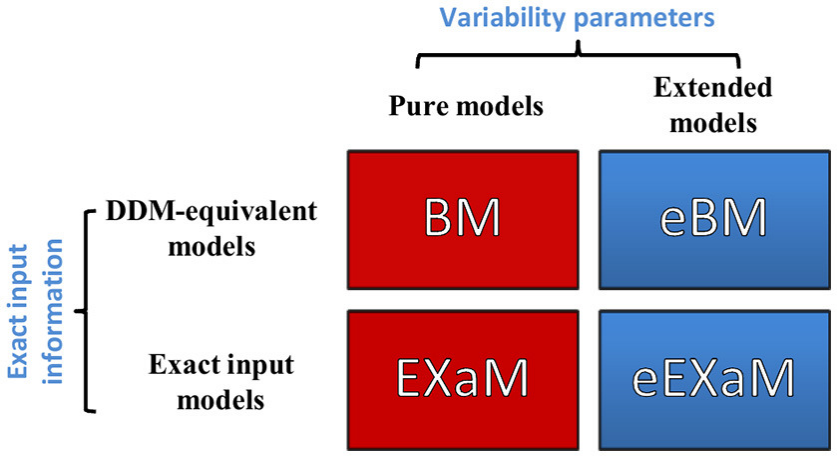
**The 2 × 2 factorial structure of model space used in this study**. The first dimension differentiates the models in terms of inter-trial variability assumptions; the pure models (red boxes) assume that the parameters are constant across trials (BM and EXaM). Adding inter-trial variability parameters leads to the extended models (blue boxes) in which the noise level and prior parameters vary across trials (eBM and eEXaM). The second dimension is about the sensory input: the DDM-equivalent models (BM and eBM) in the first row use the average information about the stimulus plus noise whereas the exact input models (EXaM and eEXaM) in the 2nd row use exact input information (see Park et al., 2016).

In the present study, we propose two novel Bayesian models for perceptual decision making in addition to two previously proposed Bayesian models (Bitzer et al., 2014; Park et al., 2016). Figure 3 shows the resulting four models with the eBM and the eEXaM as extensions of the previously proposed pure models BM and EXaM. Similarly, the exact input models EXaM and eEXaM add the processing of trial-wise stimulus features to the DDM-equivalent models BM and eBM.

## Results

Here, we validate and demonstrate the basic properties of the eBM, its close relationship to the eDDM and its usefulness for the analysis of behavioral data. In particular, we present the following results: First, we validate the theoretically-derived exact distributions of noise level and prior (Equations 10, 14) and show that the eDDM and eBM make equivalent predictions. Second, we demonstrate that the eBM with the approximate distributions still predicts the behavioral effects captured by the eDDM, specifically, the effect of task difficulty and differences in response speed between errors and correct responses. Third, we fit the eBM to responses recorded in a previous experiment (Park et al., 2016), replicate the findings from the corresponding study and show in addition that the exact input variant of the eBM, the eEXaM, explains the recorded behavior better than BM, eBM, or EXaM when the perceptual decision task is easy.

### Equivalence between eDDM and eBM using exact distributions

**Figure 4:**
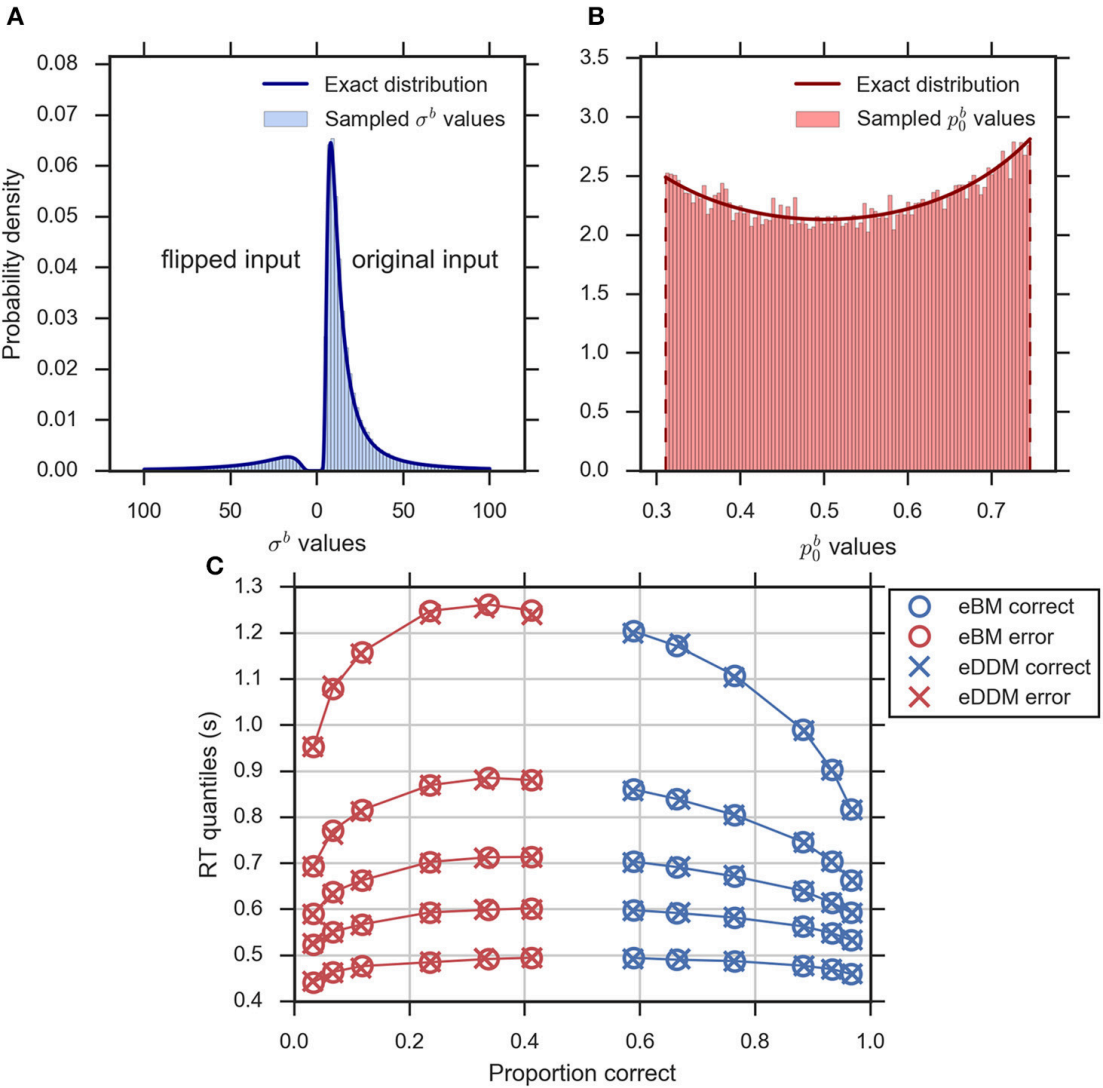
**Validity of exact equivalence equations (Equations 2–5, 10, 14). (A)** An example histogram of **σ**^*b*^ translated from the sampled eDDM ν^*d*^ values (for ${\overset{-}{\nu }}^{d}=0.133$) along with the theoretical probability density function as indicated by Equation (14). Format as in Figure 2D, **(B)** the example histogram of $p_{0}^{b}$ translated from sampled eDDM *z*^*d*^ values (for ${\overset{-}{\nu }}^{d}=0.133$) along with exact probability density function as indicated by Equation (10), **(C)** Quantile probability plot (quantiles: 0.1, 0.3, 0.5, 0.7, and 0.9) showing predicted response distributions from eDDM (crosses) and eBM (circles) based on 6 parameter sets taken from Ratcliff and McKoon (2008). RT quantiles for correct responses are shown in blue and RT quantiles for errors are shown in red. The solid lines connect the same quantile across conditions. The predictions of both models strongly agree, validating our implementations.

Equations (2–5, 10, 14) define the exact equivalence relation between eDDM and eBM. To validate specifically the exact distributions of Equations (10, 14) numerically we sampled eDDM parameters and transformed them to eBM parameters using Equations (2–5; see Section Methods for details). Figures 4A,B exemplarily shows that the exact distributions of prior and noise level, as defined in Equations (10, 14), closely match the corresponding numeric distributions.

We further validated that the eBM generates the same predictions as the eDDM when using translated eDDM parameters. To do this, we selected six previously reported parameter sets of the eDDM which only differed in the value of mean drift ${\overset{-}{\nu }}^{d}$ (Ratcliff and McKoon, 2008). For each of these parameter sets we sampled 100,000 trials for which the particular values of drift, prior and non-decision time varied according to the corresponding distributions defined by the eDDM. For each of these trials we then computed a predicted response from the eDDM producing a response time distribution from which we extracted RT quantiles for errors and correct responses. We equally produced predictions for the eBM by transforming the sampled eDDM parameters to eBM parameters using Equations (2–5) and then using our implementation of the eBM to produce RT distributions. As Figure 4C shows, the RT quantiles produced by both models coincide, as expected.

### Modeling experimental phenomena with the eBM

Here, we show that the eBM can be used as a stand-alone model to capture the same experimentally established phenomena that are typically captured well by the eDDM. To do this, we chose two well-established effects: (i) in the eDDM, behavioral differences due to task difficulty differences across conditions (e.g., coherence level in the random dot motion task) are naturally represented by differences in the mean drift rate and with a fixed drift variability. We will demonstrate that this effect can also be captured using the eBM by adjusting the mean noise level and fixing its variability. (ii) There are empirically observed patterns of relative speed of correct and error RTs, e.g., so-called slow or fast errors (Ratcliff, 1981; Ratcliff and Rouder, 1998). In the eDDM, this is typically captured by introducing a combination of the inter-trial variability of the drift rate and the inter-trial variability of the bias (Ratcliff, 1981). We will show that the combination of the equivalent parameters in the eBM, variability of noise level and variability of prior, can reproduce the same effects.

#### Modeling the effect of task difficulty

**Figure 5:**
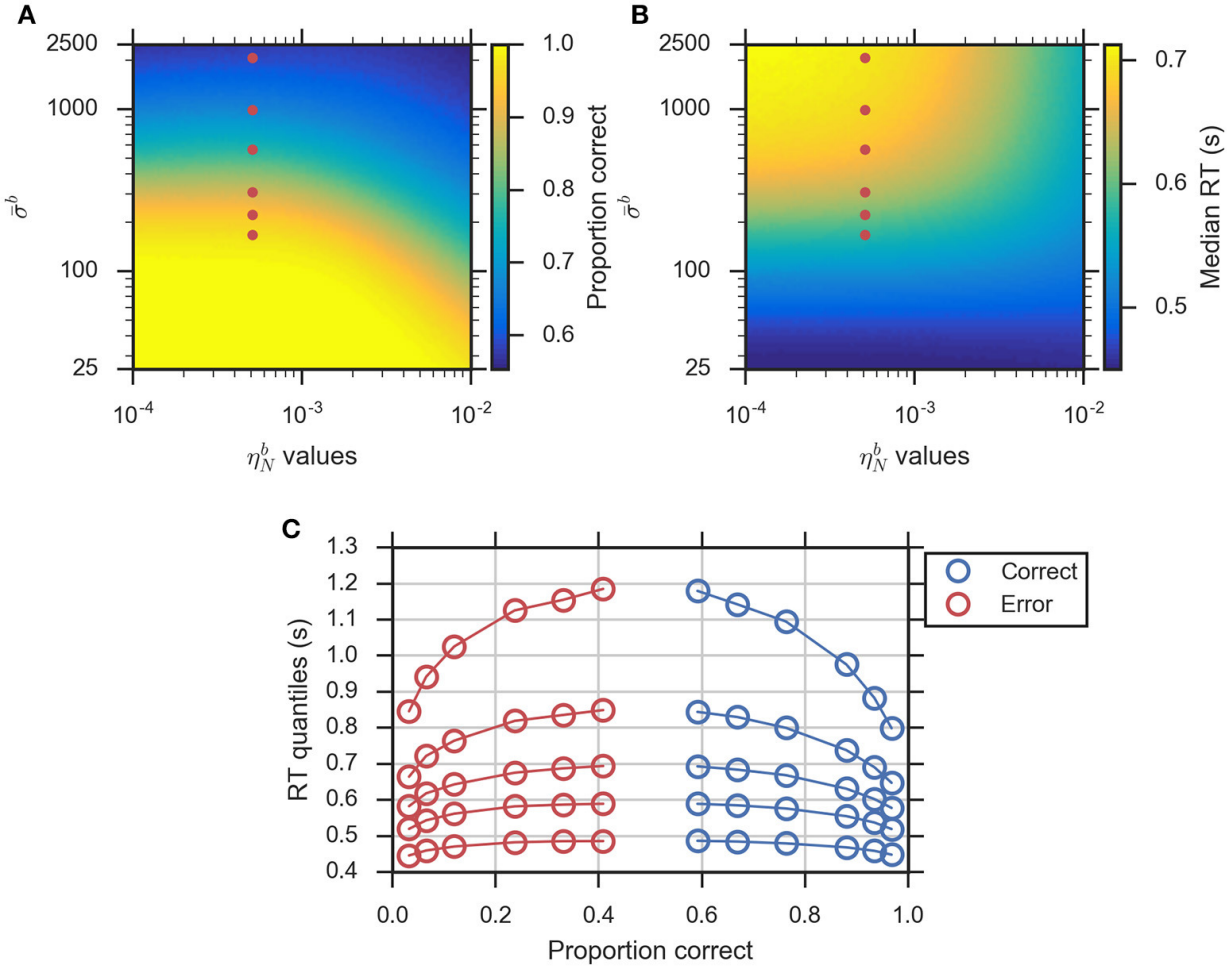
**Modeling the effect of task difficulty with the eBM. (A)** log-log plot of proportion correct and **(B)** median RT. The red filled dots indicate the exemplary combinations of (${\overset{-}{\sigma }}^{b},\eta_{N}^{b}$) selected to illustrate the effect of task difficulty using the eBM. Each of the dots represents one difficulty condition in the experiment conducted by Ratcliff and McKoon (2008), **(C)** quantile probability plot for the effect of task difficulty reproduced by the eBM: there are six conditions with correct RT quantiles (blue circles) and error RT quantiles (red circles). The solid lines connect the same quantile across conditions. As typically also found with the eDDM, as the difficulty level increases the accuracy decreases and RTs become slower. For both correct and error responses, the quantiles with long RTs are more spread out across RT.

With the eDDM, the effect of task difficulty is normally reflected by the mean drift rate (${\overset{-}{\nu }}^{d}$) which represents the speed of information uptake and is often interpreted as a measure of task performance (Philiastides et al., 2006; Ratcliff and McKoon, 2008; Voss et al., 2013; Forstmann et al., 2016). In particular, the fits of eDDM to the behavioral data for a random dot motion task show that across difficulty level (coherence percentage of random dot motion stimuli), only mean drift rates vary while the variability of the drift rate and the variability of bias have moderately large values (Ratcliff and McKoon, 2008). The equivalence equations of the eBM indicate that different noise levels should achieve the same effect as the mean drift rate in the eDDM. To show that this is the case with the approximations, we systemically varied the parameters ${\overset{-}{\sigma }}^{b}$ and $\eta_{N}^{b}$ in the range ($25<{\overset{-}{\sigma }}^{b}<2500$) and ($0.0001<\eta_{N}^{b}<0.01$). To assess the effect of these parameters, we computed the proportion correct (accuracy) and median RT for each dataset (see Section Methods for more details). We find that proportion correct (Figure 5A) varies from 0.55 to 1.0 while median RT (Figure 5B) varies between 450 and 720 ms. As expected, by increasing ${\overset{-}{\sigma }}^{b}$ and fixing $\eta_{N}^{b}$, the proportion correct decreases and median RT increases. Increasing $\eta_{N}^{b}$ has a less dramatic effect on both quantities; with $\eta_{N}^{b}>0.005$ and ${\overset{-}{\sigma }}^{b}>50$, the proportion correct decreases and median RT decreases and stays within a range of 475–600 ms. These effects are also observed qualitatively in the eDDM (see Supplementary Figure 2).

To exemplarily demonstrate how changes in task difficulty affect the response time distributions generated by the eBM, we chose six exemplary ${\overset{-}{\sigma }}^{b}$ values (168, 222, 308, 564, 986, 2075) while fixing the $\eta_{N}^{b}$ to a relatively large value (0.0005). The quantile probability plot in Figure 5C shows the RT distributions across all six datasets (100,000 trials) generated from these parameters sets. As shown in the figure, the shape of the quantile plot follows what is typically observed in corresponding experiments (see Ratcliff and McKoon, 2008 for an example) where accuracy increases with decreasing difficulty level. This is equivalent to decreasing the mean noise level, ${\overset{-}{\sigma }}^{b}$, in eBM and increasing the mean drift rate, ${\overset{-}{\nu }}^{d}$, in the eDDM. As the difficulty level increases, the median RT increases and tails of the RT distributions (the highest quantile) are spread out while there is little variation in the lowest RT quantiles, across conditions. This is in line with the general finding that changing ${\overset{-}{\nu }}^{d}$ of the eDDM will result in only a small change in the position of the lowest quantile whereas it makes a large change in the position of the highest quantile (Ratcliff and Tuerlinckx, 2002).

#### Modeling the effect of slow and fast errors

**Figure 6:**
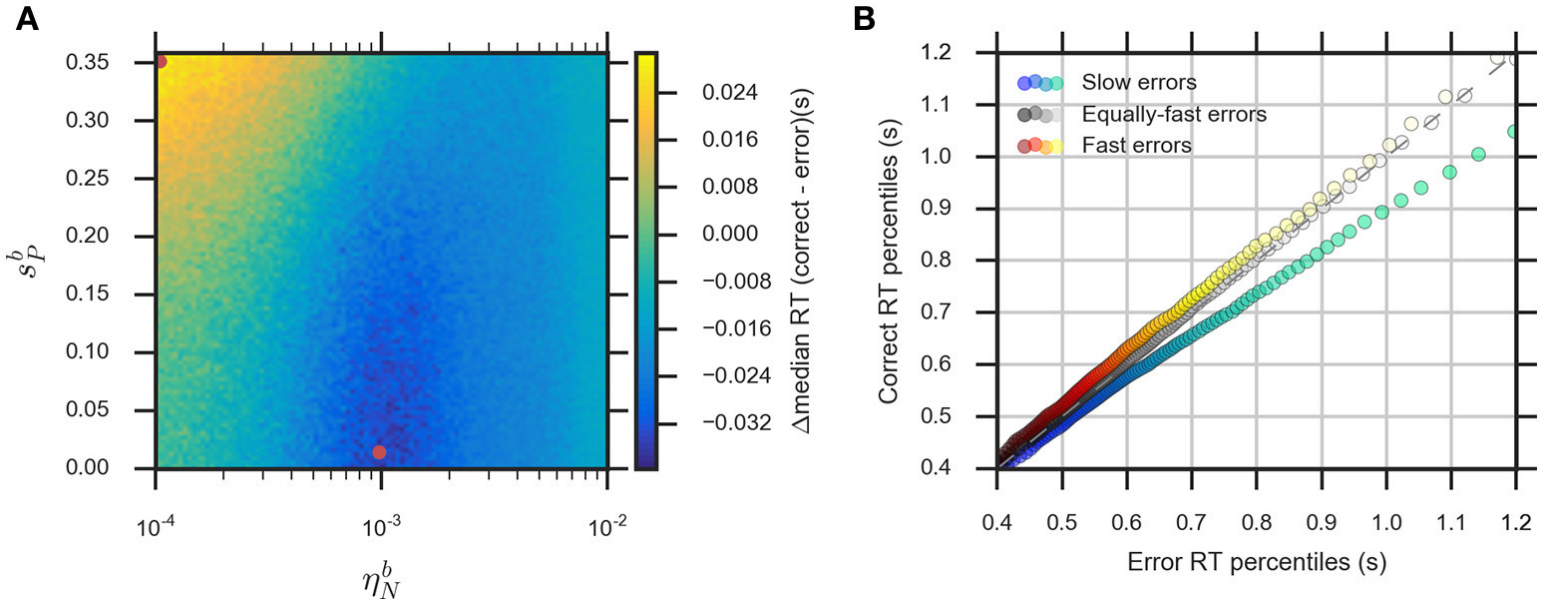
**The eBM accounts for slow and fast errors. (A)** lin-log plot of median RT difference between correct and error responses. The two red dots indicate the example parameter values chosen for demonstrating the slow and fast errors effect in **(B)** quantile-quantile plot which shows how the eBM captures either slow or fast errors. The three dotted lines depict correct and error RT percentiles (up to 100 dots per line, each dot corresponds to one of the percentiles from 1st to 100th, excluding the percentiles with very long RT) from three datasets generated by varying the values of $\eta_{N}^{b}$ and $s_{P}^{b}$. The gray dotted line represents zero variability in the noise level and prior: correct and error RTs are equal. The red-yellow dotted line above the dashed diagonal line shows the effect of fast errors (using $\eta_{N}^{b}\approx 0.0001$, $s_{P}^{b}\approx 0.35$) where the majority of error RT percentiles are faster than the ones for correct response. The blue-green dotted line below the diagonal line shows the effect of slow errors (using $\eta_{N}^{b}\approx 0.001$, $s_{P}^{b}\approx 0.014$). For all three lines, a few percentiles representing very long RTs (>1,200 ms, above 95th percentile) are not shown in the plot.

The eDDM can explain RT distributions in which errors are slower than correct responses (slow errors) by using a large variability of drift rate, η^*d*^, whereas using a large variability of bias, $s_{z}^{d}$, can generate RT distributions with fast errors (Ratcliff and Rouder, 1998; Ratcliff and McKoon, 2008; Forstmann et al., 2016). Consequently, we expect that for relatively large values of the noise variability, $\eta_{N}^{b}$, the eBM can account for slow errors, while using relatively large values for the prior variability, $s_{P}^{b}$, will lead to fast errors. In order to test this, we systematically varied the two parameters $\eta_{N}^{b}$ and $s_{P}^{b}$ in the range ($0.0001<\eta_{N}^{b}<0.01$) and $\left(0<s_{P}^{b}<0.36\right)$, while fixing the remaining parameters. We calculated the difference between the median RTs for correct and error responses (Figure 6A, see Section Methods for more details). For the used parameter ranges, the median RT difference (correct-error responses) varies from −40 to 30 ms which is in the same order of magnitude as the previously reported experimental effect (Ratcliff and McKoon, 2008). As expected, the highest median RT difference (fast errors) is obtained when we have large $s_{P}^{b}$ and low $\eta_{N}^{b}$ ($0.0001<\eta_{N}^{b}<0.0003$, $0.3<s_{P}^{b}<0.3584$), while lower values of $s_{P}^{b}$ combined with relatively large $\eta_{N}^{b}$ ($0.0006<\eta_{N}^{b}<0.002$, $0<s_{P}^{b}<0.19$) will lead to the lowest median RT differences (slow errors). With higher $\eta_{N}^{b}>0.001$, the median RT difference is negative for all possible values of $s_{P}^{b}$. A similar effect is observed with the eDDM (see Supplementary Figure 3).

We exemplarily show how the relative speed of errors and correct RT distributions are generated by the eBM, by choosing three exemplary pairs of $s_{P}^{b}$ and $\eta_{N}^{b}$ values. For generating slow errors we chose a relatively high value for $\eta_{N}^{b}\approx 0.001$ and a low value for $s_{P}^{b}\approx 0.014$ whereas we used a relatively small value for $\eta_{N}^{b}\approx 0.0001$ and a high value for $s_{P}^{b}\approx 0.35$ for generating fast errors. Also, we used a baseline parameter-set with no variability of noise and prior ($\eta_{N}^{b}=0$, $s_{P}^{b}=0$) which should generate no difference in RTs between error and correct responses. Figure 6B shows a quantile-quantile plot for RTs of correct responses in comparison to errors. In this plot, up to 100 RT percentiles (excluding only the highest percentiles with RT exceeding 1,200 ms) for correct responses are plotted against the respective percentiles for error responses to provide more detailed information on the comparison between error and correct response than the median RT provides. For those percentiles which stay above the diagonal line, the error RTs are faster than the correct RTs (cf. red-yellow line for large $s_{P}^{b}$). Similarly, percentiles below the diagonal line indicate slow errors (cf. blue-green line for large $\eta_{N}^{b}$). The slow errors effect increases for increasing percentiles. For example, the difference between the error and correct 90th percentiles (error RT: ~970 ms vs. correct RT: ~870 ms) is considerably larger than that between the 30th percentiles (error RT: 550 ms vs. correct RT: 530 ms). This is indicated by the deviation of the blue-green line downwards from the diagonal line. In contrast, the size of the fast errors effect does not strongly depend on the RT percentile. For example, the difference between correct and error 90th percentiles (error RT: ~880 ms vs. correct RT: ~900 ms) is similar to that of the 30th percentiles (error RT: ~520 ms vs. correct RT: ~540 ms). Similar effects are qualitatively observed in eDDM responses (see Supplementary Figure 3).

### Inference based on behavioral data

After validating the eBM, we will now focus on how we can use the eBM in practice to infer model parameters and to identify model versions which best explain real multi-subject behavioral data. Specifically, we will investigate to what extent the inclusion of parameter variability (extended models) and sensory input features (exact input models) can improve model fits to data (cf. Figure 3 for an overview over the considered models).

We have previously shown that the corresponding EXaM explains participant responses better than the DDM-equivalent BM (Park et al., 2016). Here, our aim is to demonstrate how the eBM can similarly benefit from exact input modeling as implemented by the eEXaM.

In particular, we focus on two model comparison questions: (1) Can we replicate the major finding of Park et al. (2016) and show that the family of exact input models (EXaM and eEXaM) can explain the behavioral data better than the family of DDM-equivalent models (BM and eBM)? and (2) Within the exact input models family, is the eEXaM a better model of participant responses than the EXaM? Especially, this second question is relevant for perceptual decision making studies, because the literature suggests that 200 trials per condition, as in our case, is insufficient to reliably infer about the variability parameters of the eDDM (Voss et al., 2013; Ratcliff and Childers, 2015). However, as our technique of exact input modeling has allowed us to infer about model parameters with higher precision before (Park et al., 2016), it appears reasonable to expect that 200 trials may be sufficient to draw informative conclusions.

To address these two questions, we re-analyzed the responses of participants from an experiment with a two-alternative forced choice task in which participants had to decide about the mean location of a single dot that jumped randomly around two targets (Park et al., 2016). The task difficulty was varied by manipulating the distance between the two targets.

We considered four models: BM, eBM, EXaM, and eEXaM. For each model, we estimated the model parameters from the behavioral data (choice and response time for each single trial) for each participant and each of four difficulty levels. For parameter inference we used EP-ABC—a Bayesian data analysis method based on simulating responses with the model and comparing them to the participant responses (Barthelmé and Chopin, 2013). EP-ABC computed the posterior parameter distribution and marginal likelihoods which we used to perform model comparison with Bayesian model selection (see Section Methods for more details).

#### Differences in parameter values

To compare the estimated model parameters across four difficulty levels for 24 participants, we computed the means of the posterior parameter distributions (see Tables 2, 3). We found two main differences when comparing the parameters fitted by the four models: The exact input models (EXaM and eEXaM) had (i) a significantly higher bound and (ii) a significantly higher variability of the non-decision time than the DDM-equivalent models. Additionally, the mean noise level, ${\overset{-}{\sigma }}^{b}$, and the mean prior, ${\overset{-}{p}}_{0}^{b}$, of the exact input models were significantly lower than those of the corresponding DDM-equivalent models in the two easiest conditions (D3 and D4).

**Table 2:** **Means of posterior parameter distributions of BM and EXaM over the four difficulty levels for 24 participants**.

**Parameter**	**BM**				**EXaM**			
	**D1 (Hard)**	**D2**	**D3**	**D4 (Easy)**	**D1 (Hard)**	**D2**	**D3**	**D4 (Easy)**
λ^*b*^	0.77 (0.01)	0.78 (0.01)	0.81 (0.01)	0.83 (0.01)	0.82^**^ (0.01)	0.84^**^ (0.02)	0.87^**^ (0.01)	0.87^**^ (0.01)
${\overset{-}{\sigma }}^{b}$	300.89 (17.90)	245.63 (12.73)	282.45 (14.00)	265.21 (10.64)	339.53 (20.71)	236.83 (13.97)	239.02^**^ (14.22)	224.26^**^ (13.09)
${\overset{-}{p}}_{0}^{b}$	0.47 (0.01)	0.47 (0.01)	0.48 (0.01)	0.48 (0.01)	0.46^*^ (0.01)	0.46 (0.01)	0.45^**^ (0.01)	0.46^**^ (0.01)
$T_{nd}^{b}$	0.55 (0.06)	0.47 (0.05)	0.43 (0.04)	0.38 (0.04)	0.55 (0.05)	0.47 (0.04)	0.42 (0.03)	0.41^*^ (0.04)
$s_{t}^{b}$	0.41 (0.06)	0.33 (0.04)	0.27 (0.04)	0.22 (0.04)	0.51^**^ (0.05)	0.45^**^ (0.04)	0.40^**^ (0.03)	0.36^**^ (0.03)
$\pi_{l}^{b}$	0.03 (0.01)	0.03 (0.01)	0.02 (0.00)	0.02 (0.00)	0.02 (0.00)	0.03 (0.01)	0.02 (0.00)	0.03 (0.01)
$\pi_{to}^{b}$	0.34 (0.03)	0.32 (0.03)	0.35 (0.02)	0.28 (0.02)	0.36 (0.02)	0.28 (0.03)	0.36 (0.03)	0.30 (0.03)

Shown are the means over participants and the corresponding standard error in parentheses. The bound (λ^b^) and the prior mean (${\overset{-}{p}}_{0}^{b}$) are both in terms of probabilities (ranging from 0 to 1). A prior mean of 0 indicates a complete bias toward the right choice and 1 a complete bias toward the left one. Asterisks indicate a significant difference between EXaM and BM parameters for each condition (

* *p < 0.05*,

** *p < 0.01, based on a paired t-test over 24 participants). See also Table 1 for the meaning of parameters*.

**Table 3:** **Means of posterior parameter distributions of eBM and eEXaM**.

**Parameter**	**eBM**				**eEXaM**			
	**D1 (Hard)**	**D2**	**D3**	**D4 (Easy)**	**D1 (Hard)**	**D2**	**D3**	**D4 (Easy)**
λ^*b*^	0.77 (0.01)	0.79 (0.01)	0.82 (0.01)	0.84 (0.01)	0.82** (0.01)	0.85** (0.02)	0.88** (0.01)	0.88** (0.01)
${\overset{-}{\sigma }}^{b}$	320.56 (21.82)	249.12 (13.39)	273.18 (15.84)	256.04 (11.29)	331.77 (22.47)	224.82 (14.98)	231.46* (13.59)	205.72** (12.36)
$\eta_{N}^{b}$	0.0028 (0.0022)	0.0009 (0.0003)	0.0006 (0.0004)	0.0004 (0.0001)	0.0004 (0.0002)	0.0005 (0.0002)	0.0003 (0.0001)	0.0005 (0.0001)
${\overset{-}{p}}_{0}^{b}$	0.47 (0.01)	0.46 (0.01)	0.48 (0.01)	0.47 (0.01)	0.46* (0.01)	0.46 (0.01)	0.46** (0.01)	0.46** (0.01)
$s_{P}^{b}$	0.06 (0.01)	0.05 (0.00)	0.05 (0.00)	0.05 (0.01)	0.05 (0.01)	0.05 (0.00)	0.05 (0.01)	0.05 (0.00)
$T_{nd}^{b}$	0.56 (0.06)	0.48 (0.05)	0.43 (0.04)	0.39 (0.04)	0.55 (0.05)	0.47 (0.04)	0.42 (0.03)	0.42* (0.04)
$s_{t}^{b}$	0.42 (0.06)	0.34 (0.04)	0.28 (0.04)	0.23 (0.04)	0.51* (0.05)	0.46** (0.04)	0.40** (0.03)	0.36** (0.03)
$\pi_{l}^{b}$	0.03 (0.01)	0.03 (0.00)	0.02 (0.00)	0.02 (0.00)	0.02 (0.00)	0.03 (0.00)	0.02 (0.00)	0.02 (0.01)
$\pi_{to}^{b}$	0.38 (0.03)	0.34 (0.02)	0.40 (0.02)	0.33 (0.02)	0.36 (0.02)	0.30 (0.02)	0.37 (0.02)	0.34 (0.03)

*Format as in Table 2, see also Table 1 for the meaning of parameters*.

#### Model comparison

**Figure 7:**
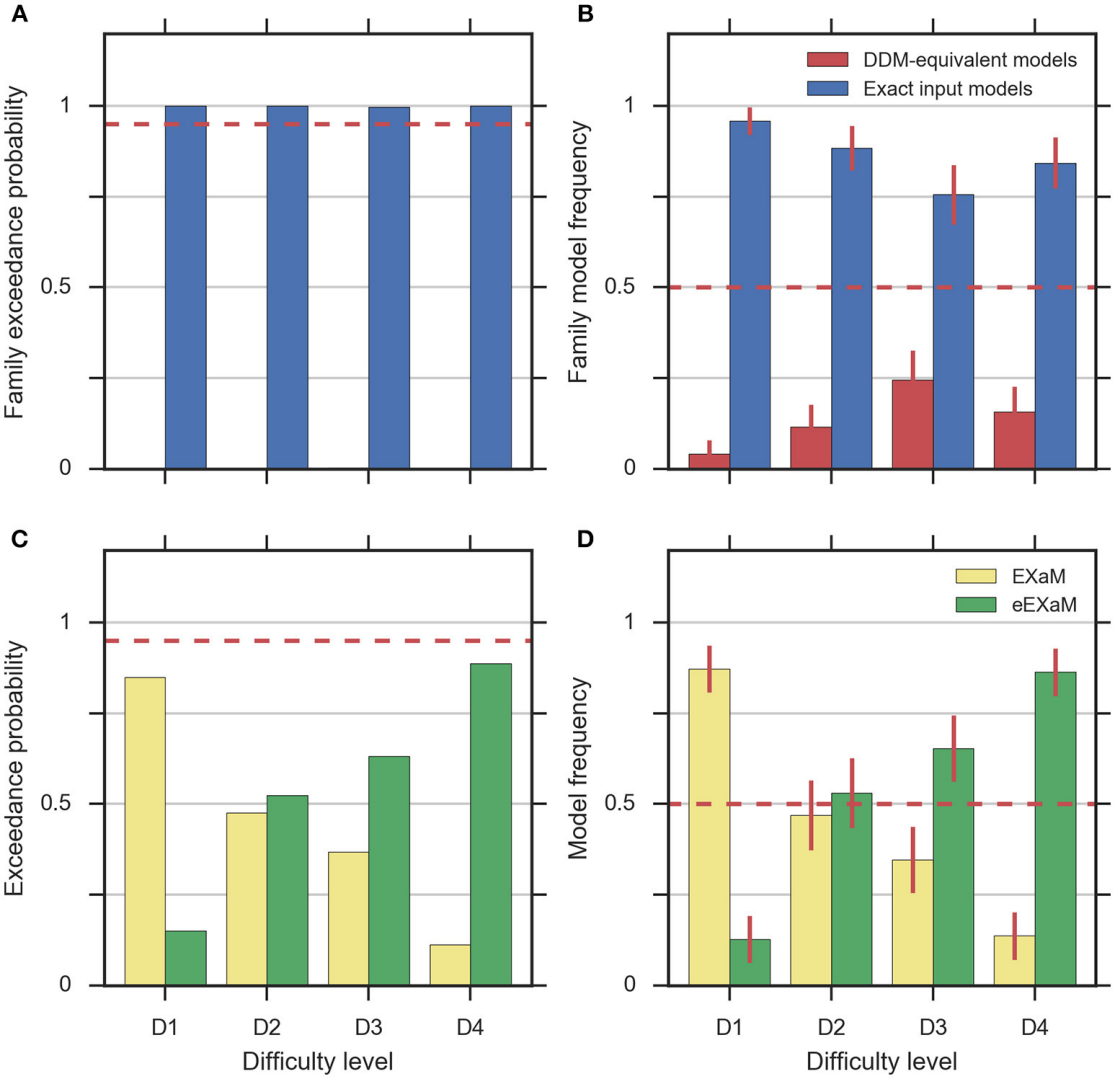
**Results of random-effects Bayesian model comparison across four difficulty conditions for 24 participants, (A)** protected family exceedance probability and **(B)** family model frequency for family inference between exact input models and DDM-equivalent models. **(C)** Protected exceedance probability and **(D)** model frequency for model comparison between EXaM and eEXaM. Protected exceedance probability is the estimated probability that the given family of models wins the model comparison in the population of participants. For **(A,C)** the red dashed line represents the threshold indicating very strong evidence for a model (0.95). The model frequency refers to the probability that a randomly selected participant behaves according to the considered family of models. For **(B,D)**, the red dashed line indicates chance level and error bars indicate the posterior standard deviation of the estimated model frequencies.

To address the question whether exact input models better explain participant responses than the DDM-equivalent models we conducted family model inference (Penny et al., 2010; Daunizeau et al., 2014) for comparing the family of exact input models (EXaM and eEXaM) and the family of DDM-equivalent models (BM and eBM). Model comparison was performed by computing the protected exceedance probabilities and model frequencies (i.e., posterior model probabilities; Stephan et al., 2009; Daunizeau et al., 2014; Rigoux et al., 2014) based on the marginal likelihoods returned by EP-ABC. Addressing our first question, we found strong evidence that exact input models (EXaM, eEXaM) better explain the participants' responses than DDM-equivalent models (BM, eBM): The family protected exceedance probability was ~1.00 for all difficulty levels (Figure 7A) which means that the belief that the exact input models are more likely than the DDM-equivalent models is nearly 100%. The family model frequency was on average above 75% (Figure 7B) which means that the probability that the exact input models generated the data for any randomly chosen participant is above 75%. These results are in congruence with the findings by Park et al. (2016) that modeling the exact input presented to the participants leads to a better model than using the average input information in the DDM-equivalent models.

To investigate our second question we conducted a 2-way model comparison between the EXaM and the eEXaM. Figures 7C,D shows that neither of these two models wins across all conditions. Rather, EXaM outperforms eEXaM in the most difficult condition (D1) whereas the reverse is true in the easiest condition (D4). Specifically, the protected exceedance probability (Figure 7C) is nearly 85% for EXaM in D1, and above 88% for eEXaM in D4. Similarly, the model frequency (Figure 7B) shows the same effect: In D1 the model frequency for EXaM is above 87% and in D4 the model frequency for eEXaM is above 86%. Furthermore, exceedance probabilities and model frequencies reach intermediate values in the intermediate difficulty conditions (D2 and D3) indicating that the two models cannot be discriminated clearly in these conditions. Across all conditions, Figures 7C,D suggests that there is a gradual relationship between the usefulness of parameter variabilities in explaining participant responses and the difficulty of the decisions: As decisions become easier the eEXaM becomes more likely.

In addition, we investigated whether we find the same trend of results when conducting a 2-way model comparison between the DDM-equivalent models (BM and eBM). However, the results look very different: the protected exceedance probability is above 89% for the BM in the D1 to D3 conditions (Supplementary Figure 4A), and nearly 74% for the D4 condition. In analogy, the model frequency is above 75% for BM in all conditions (Supplementary Figure 4B). These results show that when not using the exact input, there is not much evidence, given these data, for additional inter-trial parameter variability. This finding is in stark contrast to what we found for the exact input models and may hint at why the literature suggests that variability parameters cannot be determined reliably, see Discussion.

## Discussion

We have derived an eBM for perceptual decision making by translating commonly used extensions of the DDM to an equivalent Bayesian formulation. Specifically, we have shown how the inter-trial variability of the parameters “noise level” and “prior” in a Bayesian model can emulate the corresponding variability of “drift” and “bias” parameters in the extended drift diffusion model. To mathematically simplify the resulting extended Bayesian (eBM) model we proposed approximations for the derived parameter distributions and showed that the resulting model reproduces key behavioral effects reported in the literature, such as the effect of task difficulty, and differences in response time of correct responses and errors. We further demonstrated the practicality of the eBM for the analysis of experimentally observed response behavior by re-analyzing a dataset from recent work (Park et al., 2016). Crucially, for the analysis of this dataset, we provided the known trial-wise spatiotemporal details of the stimulus to the eBM. Similar to previous findings (Park et al., 2016), our results showed that this exact input model provides for a better model. Only when using exact input models, we found support for the additional inter-trial variability of the eBM. This was not the case when not using exact input models: in this case we found that the best model is the pure DDM-equivalent model, without using the additional variability parameters.

The here presented exact input models extend the line of work on including spatiotemporal details of the stimulus into model-based analyses of decision making behavior (Brunton et al., 2013; Park et al., 2016). In accordance with previous results (Park et al., 2016), group-level model comparison (Figure 7) indicated that the exact input models (EXaM and eEXaM) explain choice and response times better than the DDM-equivalent models without such exact input (BM and eBM). Furthermore, the fitted values for the “bound” parameter were significantly higher in the exact input models compared to the DDM-equivalent models (Tables 2, 3). This suggests that the exact input models explained a larger portion of response times using the evidence accumulation mechanism as compared to the DDM-equivalent models, because with a larger bound decisions tend to be slower and a smaller portion of the participant's response times remains to be explained as non-decision time. Both findings together strongly suggest that the exact input models are better models of the decision making process than the DDM-equivalent models.

Using exact input models not only lead to a general increase in the explanatory power of the models, but also increased the sensitivity of model comparison between pure and extended models. Using the stimulus features as input to the models, we found evidence that the extended EXaM (eEXaM), which includes inter-trial parameter variability, explains participant responses better than the EXaM, which uses constant parameters, when decision are easy (Figure 7). In contrast, we did not find corresponding evidence with the DDM-equivalent models which only use average stimulus information as input (Supplementary Figure 4): The BM by far outperforms the eBM in all conditions.

The observed increase in sensitivity of the exact input models for using additional variability parameters demonstrates the power of including within-trial changes of stimulus information into perceptual decision making models. Especially, it has previously been reported that inter-trial variability parameters of the eDDM can only be identified from large datasets (Voss et al., 2013; Ratcliff and Childers, 2015). With the eEXaM, however, we find evidence for inter-trial parameter variability in a moderately-sized dataset with 200 trials per participant and condition. This improvement of explanatory power over the standard DDM may be crucial for differentiating the computational mechanisms underlying perceptual decision making behavior (Brunton et al., 2013; Park et al., 2016). This explanatory power may also enable novel model-based neuroimaging studies for studying the neural basis of perceptual decision making and related underlying mechanisms (Philiastides et al., 2006; Frank et al., 2015; Turner et al., 2015).

**Figure 8:**
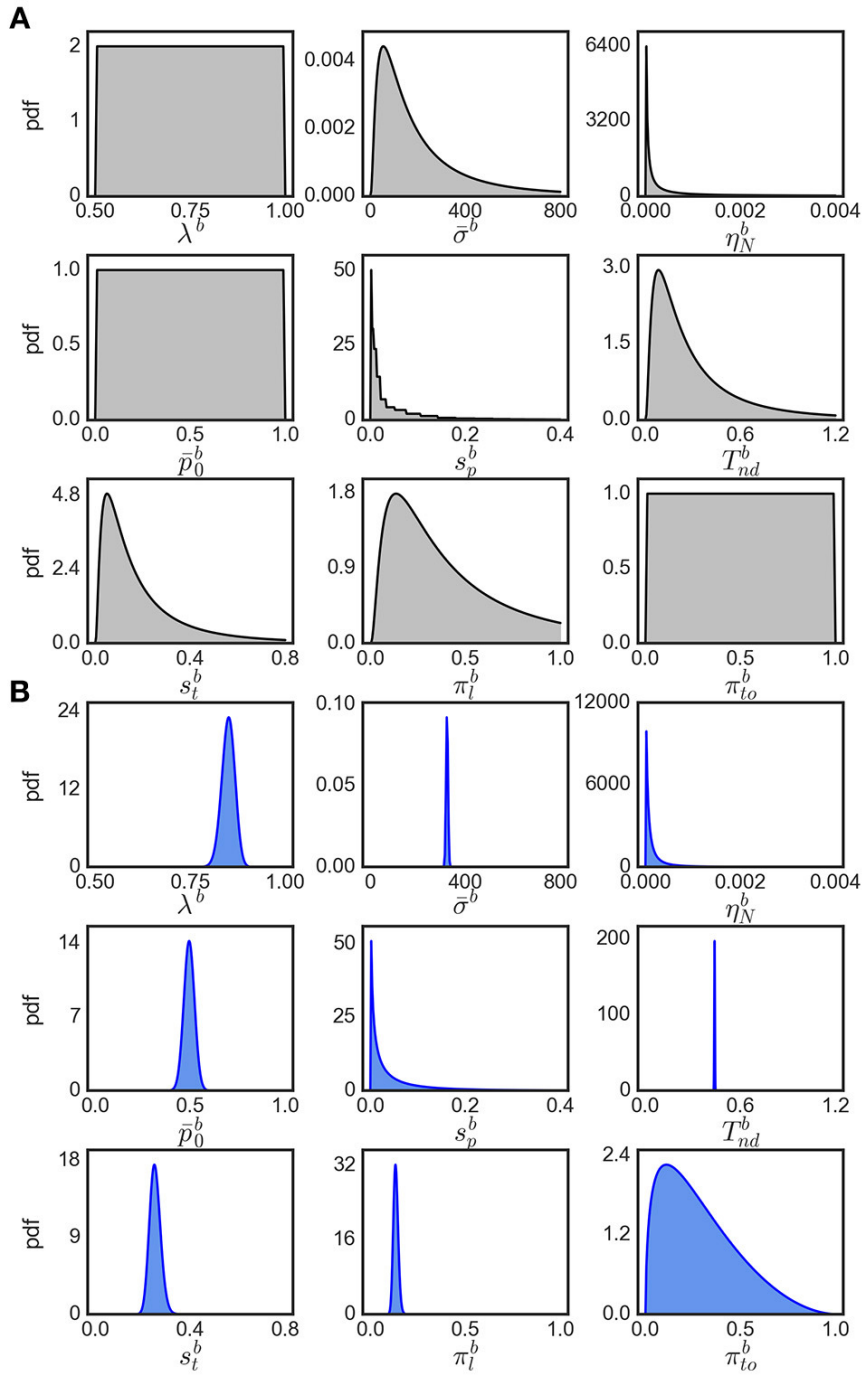
**Visualization of parameter distributions. (A)** Prior densities for each parameter in Table 4, **(B)** example (marginal) posterior parameter densities of the eBM for participant 17, condition 40.

Note that the evidence for variability parameters in exact input models is based on model comparison and is not necessarily related to the ability to identify particular values for these parameters. In fact, we found that the inferred posterior distributions over variability parameters are almost as wide as the corresponding priors suggesting that these parameters could not be identified well (see Figure 8 for an example). However, using model comparison we can state that some parameter variability in the considered ranges is beneficial for modeling the responses for easy decisions, but we cannot reliably pin down a specific amount of parameter variability that would best fit to the responses.

Enriching decision models with spatiotemporal details of the stimulus is not limited to Bayesian decision models. Brunton et al. (2013) demonstrated how a DDM can be used to model stimulus fluctuations in the form of brief pulses. The equivalence between the DDM and a corresponding Bayesian model (Bitzer et al., 2014) suggests that more general, continuous stimulus fluctuations can also be incorporated into DDMs. In fact, Equation (13) states that the drift rate of a DDM depends on the mean stimulus feature value currently presented to the subject ($\mu_{i\left(r\right)}^{b}$). For the single dot task considered here, this prescribes that the drift of a corresponding DDM should equal to the scaled x-position of the currently presented dot, as pointed out by one of our reviewers. Similarly, stimulus features may modulate energy landscapes of attractor models (see Wang, 2002; Insabato et al., 2014). The additional advantage of a Bayesian formulation is that it provides for a formally rigorous connection between raw stimulus features and updates of a decision variable and, thus, provides the not necessarily intuitive computation for how spatiotemporal details of the stimulus affect the decision, also for potentially more complex stimuli such as multi-sensory stimuli.

**Table 4:** **Inferred parameters in the models with their prior distributions**.

**Parameter**	**Prior**	**Prior Parameters**	
		**Mean**	**Std**
λ^*b*^	Uniform	0	1
${\overset{-}{\sigma }}^{b}$	Log-normal	5	1
$\eta_{N}^{b}†$	Log-normal	−10	3
${\overset{-}{p}}_{0}^{b}$	Uniform	0	1
$s_{P}^{b}†$	(cf. Figure 8A)	−2	1
$T_{nd}^{b}$	Log-normal	−1.5	1
$s_{t}^{b}$	Log-normal	−2	1
$\pi_{l}^{b}$	(cf. Figure 8A)	−1	1
$\pi_{to}^{b}$	Uniform	0	1

*The parameters indicated by † are the ones specific to the extended models (eBM and eEXaM)*.

We found that the eEXaM became increasingly the better model than the EXaM in explaining participant responses as the decisions became easier (Figures 7C,D). Although, striking, we can only speculate about a possible cause for this finding: one possible reason might be that the additional inter-trial parameter variabilities in the eEXaM equip the model with the ability to vary the strength with which a stimulus affects the decision across trials. That is, in one trial a participant may follow the stimulus quite closely while in another only casually and the eEXaM can capture this by allowing the noise level to vary across trials. This mechanism, however, appears to interact with the difficulty of the decision as it only appears to be important for easy decisions. We speculate that this is because in hard decisions the model already has to use relatively high noise levels to explain responses such that the additional variability of the noise level does not significantly contribute to explaining the responses. This is supported by our parameter inference results (Tables 2, 3) where we found that the mean noise level decreased from hard to easy decisions in the EXaM and eEXaM while the particular values were similar between both models for any given difficulty.

We demonstrated the applicability of the eEXaM to a two-alternative forced choice paradigm which is based on a specific single dot stimulus (Park et al., 2016), but the model also applies to other paradigms. For example, the model can be easily modified to include multi-alternative decisions by adding internal generative models (cf. Figure 1) for each additional decision alternative. The eEXaM may also be applied to any stimulus for which a sequence of feature values can be extracted which describes the evolution of the stimulus within a trial. For the common random dot motion stimuli such feature values may be the so-called motion energy (Kiani et al., 2008) at a given time point. Further extension of the model for multidimensional stimuli, for example, comprising of an auditory and a visual dimension are conceivable as well. Allowing for different noise levels and internal uncertainties in the different dimensions would then enable one to study dynamic effects of multi-sensory integration in response time distributions.

We provided approximations for exact probability distributions Equations (10, 14) to make the eBM formulation independent from the eDDM parameters. Despite our efforts to make the eBM equivalent to the eDDM, there are conditions in which we expect our approximations to introduce slight deviations from the eDDM (i.e., the approximated distributions deviate from exact distributions). Specifically, when the distribution of the prior in the eBM is wide or the mean prior is far from 0.5, the approximated distribution of the prior (Equation 15) deviates more strongly from the exact distribution (Equation 10). Also, the approximated distribution of the noise level (Equations 17, 19) differs more from the corresponding exact distribution (Equation 14), when the skewness of the inverse Gaussian becomes larger i.e., when there is large variability of noise level in relation to its mean. However, we expect that these deviations do not lead to relevant differences in behavioral predictions of eBM and eDDM in typical experimental conditions, because the behavioral effects of parameter variability are small and it has been previously found that the eDDM is relatively robust against changes in the precise shape of the parameter distributions (Ratcliff, 2013).

In the present work we have chosen to relate drift rate variability in the DDM to noise level variability in the BM based on Equations (2–5). However, we have previously also presented an alternative parameterization of the BM in which drift relates to the mean input instead to the noise level (Bitzer et al., 2014, Equations 16–19). Our main motivation for not using this parameterization is that it does not correspond well to the situation in the single dot task experiment (Park et al., 2016). In particular, we assume that the mean input μ^*b*^ is constrained by the actual dot positions and we did not want to mix this effect with subject-specific decision making properties. Consequently, we chose the present parameterization in which DDM-drift translates to a subject-specific modulation of the noise around the stimulus-driven (exact) mean input.

The core decision making mechanism implemented by the DDM rests upon two effective parameters which are often chosen to be drift and bound while the diffusion coefficient is arbitrarily fixed. In the BM we also use 2 effective parameters: noise level (σ^*b*^) and bound while fixing the internal uncertainty (${\hat{\sigma }}^{b}$), to a value derived from σ^*b*^ (Equation 6). We included the remaining parameters (related to lapses, non-decision time and bias), because we have found in previous work that they improve fits of the model to behavioral data (Park et al., 2016).

Note that the work proposed here differs from Bayesian schemes which have been used to estimate the parameters of the DDM from behavioral data (Wiecki et al., 2013) as it uses Bayesian inference to model the actual decision mechanisms opposed to using Bayesian inference just for data analysis. The eBM is proposed in line with other models that formalize the perceptual decision making process as noisy accumulation of sensory observations (Dayan and Daw, 2008; Drugowitsch et al., 2012). The eBM conceptually connects to predictive coding (Rao and Ballard, 1999) by interpreting the decision making process as comparison of top-down predictions to the observed sensory input (Summerfield et al., 2006; Bitzer et al., 2014, 2015; Summerfield and de Lange, 2014). Further, the eBM quantifies decision making behavior using an estimate of the subjective, internal uncertainty of the decision maker about his or her sensory observations. These properties of the eBM and other Bayesian models have been found beneficial in developing computational descriptions of brain function (Friston, 2010; Pouget et al., 2013).

## Methods

### The bayesian model (BM)

In this section, we will briefly describe the Bayesian model (BM) used as the core of eBM in this paper (Bitzer et al., 2014). The BM consists of (1) sensory input process, (2) generative models for each alternative, (3) the evidence accumulation process with Bayesian inference, (4) decision policy. The first component is the input model while the remaining three components implement the decision model.

#### The sensory input process

The decision making process in the BM is based on encoding the time-dependent features of the sensory input that are used to compute the evidence for each alternative (Figure 1i). The BM can use the exact information about the stimulus as input to the decision model (Bitzer et al., 2014; Park et al., 2016). For example, this information can be the location of dots on the screen. In the BM, the sensory stimulus is represented by the feature values *x*_*t*_ which are assumed to be distributed according to a Gaussian distribution:

(20) $$\begin{array}{l} x_{t}\sim N\left(\mu_{i\left(j\right)}^{b},\Delta t\sigma^{b^{2}}I\right) \end{array}$$

where $\mu_{i\left(j\right)}^{b}$ refers to the mean feature values for the *i*-th alternative and *j*-th trial, *I* is the identity matrix, and Δ*tσ^b^*^2^ is the variance of the input noise. Note that the BM can incorporate sensory features with a dimension higher than one.

#### The generative models

The generative models (Figure 1ii) in the BM are used to compute the internal belief of the decision maker about the observed stimulus. For each alternative, *A*_*i*_, there exists a generative model that maps the abstracted observation, *x*_*t*_, to a probability density value of that observation given that alternative, *p*(*x*_*t*_ |*A*_*i*_) as a Gaussian density function:

(21) $$\begin{array}{l} p\left(x_{t}|A_{i}\right)=N\left({\hat{\mu }}_{i}^{b},\Delta t{\hat{\sigma }}^{b^{2}}I\right) \end{array}$$

where ${\hat{\mu }}_{i}^{b}$ is the mean feature value for the i-th alternative and ${\hat{\sigma }}^{b}$ is the internal uncertainty of the sensory observations. Note that ${\hat{\sigma }}^{b}$ is determined by the sensory input noise, σ^*b*^, based on Equation (6).

#### The evidence accumulation process

The model accumulates the evidence for an alternative by using Bayesian inference recursively (Figure 1iii). This process starts from a prior probability of each alternative, *p*(*A*_*i*_). At each time-step *t*, the model computes the posterior belief, p(*A*_*i*_|*X*_1:*t*_), that each alternative is true given the sensory inputs over time:

(22) $$\begin{array}{l} p\left(A_{i}|X_{1:t}\right)=\frac{p\left(x_{t}|A_{i}\right)p\left(A_{i}|X_{1:t-1}\right)}{\sum_{j\;=\;1}^{M}p\left(x_{t}|A_{j}\right)p\left(A_{j}|X_{1:t-1}\right)} \end{array}$$

where *M* is the number of alternatives and *X*_1:*t*_ = {*x*_1_, *x*_2_, …, *x*_*t*_} is a set of sequentially determined sensory observations up to the current time-step.

#### The decision policy

The decision policy uses the computed posterior belief *p*(*A*_*i*_|*X*_1:*t*_), as the decision variables of the model, to account for decision criteria (Figure 1iv). In the BM, a decision is made when one or more of these posterior beliefs reach a set bound, λ^*b*^. Consequently, the selected alternative is the one with the maximum posterior belief that exceeds the bound:

(23) $$\begin{array}{l} \underset{i}{\max}p\left(A_{i}|X_{1:t}\right)\geq \lambda^{b} \end{array}$$

### Modeling lapses

In previous work (Park et al., 2016), we equipped the BM with the ability to explain a response as random lapse. This makes the model robust against outliers during fitting of data. We followed similar approaches in the literature (Drugowitsch et al., 2012) and modeled lapses as responses with random choice and uniform response time distribution *U*(0, *maxrt*^*b*^) where *maxrt*^*b*^ is the maximum allowed response time. The model selects a trial as a lapse trial with “lapse probability,” $\pi_{l}^{b}$. To model lapses in which the response of the participant is timed out the model additionally selects lapses which become timed-out with a probability $\pi_{to}^{b}$. Therefore, the model will produce a timed-out lapse trial with probability $\pi_{l}^{b*}\pi_{to}^{b}$.

### Parameter sweeps

To demonstrate how the eBM captures the task difficulty and slow and fast errors, we used parameter sweeps over the selected eBM parameters. For modeling the task difficulty effect (Figures 5A,B), we varied the two parameters ${\overset{-}{\sigma }}^{b}$ and $\eta_{N}^{b}$ in the range ($25<{\overset{-}{\sigma }}^{b}<2500$, $0.0001<\eta_{N}^{b}<0.01$) while fixing the remaining parameters (λ^*b*^ = 0.8, ${\overset{-}{p}}_{0}^{b}=0.5$, $s_{P}^{b}=0.2$, $T_{nd}^{b}=0.4$, $s_{t}^{b}=0.2$, $\pi_{l}^{b}=0$, $\pi_{to}^{b}=0$). For modeling the slow and fast errors effect (Figure 6A), we varied the two parameters $\eta_{N}^{b}$ and $s_{P}^{b}$ parameters in the range ($0.0001<\eta_{N}^{b}<0.01$, $0<s_{P}^{b}<0.3584$), while fixing the remaining parameters (λ^*b*^ = 0.8, ${\overset{-}{\sigma }}^{b}=222.5538,\;{\overset{-}{p}}_{0}^{b}=0.5$, $T_{nd}^{b}=0.4$, $s_{t}^{b}=0.2$, $\pi_{l}^{b}=0$, $\pi_{to}^{b}=0$). For both parameter sweeps, we set the eBM mean feature values to $\mu_{1}^{b}=25$ and $\mu_{2}^{b}=-25$ and used Δ*t* = 0.05. We simulated a dataset with 100,000 decisions for each parameter-set. The responses with RT bigger than 5,000 ms were marked as timed-out trials. Finally, we calculated the proportion correct and median RT (for task difficulty, see Figures 5A,B), and the difference between median RT for correct and error responses (for slow and fast errors, see Figure 6A).

### Data of Park et al. (2016)

The experiment used a novel behavioral paradigm for studying perceptual decision making. This paradigm consists of a two-alternative forced choice task with four difficulty conditions (200 trials each) presented in a randomized order. In each trial, after a random fixation period (300–500 ms), two yellow targets, left and right from the screen center, appeared on the screen (for 700 ms). After this target presentation period, a white dot started jumping randomly around the two targets. The location of the white dot changed every 93.2 ms and was shown until the participant made a decision up or until a maximum of 25 iterations (2.33 s) after which the response was timed out. The task had four difficulty levels in which the horizontal location of the target positions differed: Targets were either 55 (easy), 40, 25, or 10 (hard) pixels left or right, respectively, from the center of the screen. The visual stimuli were generated before the experiment and every participant was presented with the same set of rendered dots although in a different, randomized order of presentation. The time-dependent positions of the dots were stored and used for fitting the exact input models.

### Inference over models given behavioral data

We used EP-ABC (Barthelmé and Chopin, 2013) for Bayesian inference of model parameters and estimation of the marginal likelihood for subsequent model comparison. This method combines a Monte-Carlo approach to inference (ABC) with a variational Gaussian approximation of parameter posterior distributions (EP). Specifically, EP-ABC does not require an analytic definition of the likelihood of the model and instead only relies on simulations from the considered model. For details of this method see Barthelmé and Chopin (2013).

For our analyses here we used a Python implementation of EP-ABC that is available at http://github.com/sbitzer/pyEPABC. We independently ran EP-ABC for each of the four models (BM, eBM, ExaM, and eEXaM), for each condition (D1:Hard, D2, D3, and D4:Easy) and each of the 24 participants, leading to 384 runs of EP-ABC in total. The data set in each run comprised of the choices and RTs for the 200 trials of the particular chosen participant and condition. We used seven free parameters for the basic models with two extra variability parameters for the extended models (Table 4). For each participant and each difficulty level, the behavioral data (RT and choice responses) are used to estimate the parameters.

Being a Bayesian method, EP-ABC requires the definition of a prior distribution over model parameters. Specifically, our implementation of EP-ABC assumes a multivariate Gaussian prior and returns a multivariate Gaussian posterior. To allow non-Gaussian distributions, especially, distributions which are restricted to certain value ranges, we used two parameter transformations as in Park et al. (2016): (1) exponential transformation, and (2) uniform transformation. The exponential transformation maps a real value to a positive real value. Thus, it transforms a Gaussian distribution into a log-normal distribution. The uniform transformation maps a real value through the cumulative Gaussian density function and then scales and shifts the values further. Thus, it transforms a standard normal distribution to a uniform distribution in a desired range, but can implement biases toward certain regions in this range when the transformation is applied to a different Gaussian distribution.

We assume that the parameters are a priori uncorrelated and, consequently, set the covariance of parameters in the prior to 0. Each parameter prior is then defined by a univariate Gaussian distribution potentially together with a transformation. The particular parameter prior settings we used in our analyses are shown in Table 4 and the resulting univariate prior distributions are depicted in Figure 8A.

EP-ABC itself has parameters which trade off the computational burden of the method with the quality of the approximate Bayesian inferences. For our analysis we selected parameters focusing on the quality of the inference. We set the acceptance threshold to ε = 0.05 which means that a sampled response was accepted when it had the same choice as the participant in that trial and the RTs differed by no more than 0.05 s. The minimum number of accepted samples was 2,000, the maximum number of samples per trial was 6,000,000, alpha was 0.5, veps was 0.2 and we passed through the data twice. See Barthelmé and Chopin (2013) and the documentation of EP-ABC at http://github.com/sbitzer/pyEPABC for interpretation of these parameters.

The output of EP-ABC is a multivariate Gaussian posterior distribution as well as the marginal likelihood of the model. To get parameter values in their original space defined by the model, we sample from the posterior distribution and transform the samples through the associated functions. The reported posterior means (Tables 2, 3) are the means of the transformed samples. An example of univariate slices of the full posterior probability density is illustrated in Figure 8B.

### Bayesian model selection

To formally compare the models, we used the random-effect Bayesian model selection (RFX-BMS) procedure (Stephan et al., 2009; Daunizeau et al., 2014; Rigoux et al., 2014) which computes the protected exceedance probability and model frequency(on family level or 2-way model comparison) based on the marginal log-likelihood of produced by EP-ABC method. The RFX-BMS procedure was applied using the Variational Bayesian Analysis (VBM) toolbox, as available at: http://sites.google.com/site/jeandaunizeauswebsite/code/rfx-bms.

## Ethics statement

The experimental procedure was approved and carried out in accordance with the guidelines by the ethics committee of the University of Leipzig (Park et al., 2016).

## Author contributions

PF, AW, SK, and SB designed the study. HP acquired the data. PF, SB, and SK analyzed the data. PF, HP, AW, SK, and SB wrote the manuscript.

## Funding

This work was supported by the Deutsche Forschungsgemeinschaft (SFB 940/2, Project Z2) and the Open Access Publication Funds of the TU Dresden.

### Conflict of interest statement

The authors declare that the research was conducted in the absence of any commercial or financial relationships that could be construed as a potential conflict of interest.
