# Supplementary material for: A Bayesian Reformulation of the Extended Drift-Diffusion Model in Perceptual Decision Making
Source: Front Comput Neurosci. 2017 May 11;11:29. doi: 10.3389/fncom.2017.00029 (PMC5425616; doi:10.3389/fncom.2017.00029)
Supplement: Supplementary file 1 [file DataSheet1.PDF]

Supplementary Material

A Bayesian reformulation of the extended drift-diffusion model in perceptual decision making

Pouyan R. Fard[1]*, Hame Park[1], Andrej Warkentin[2], Stefan J. Kiebel[1], Sebastian Bitzer[1]

[1]Department of Psychology, Technische Universität Dresden, Dresden, Germany

[2]Bernstein Center for Computational Neuroscience, Berlin, Germany

*** Correspondence:**
Pouyan R. Fard
pouyan.fard@tu-dresden.de

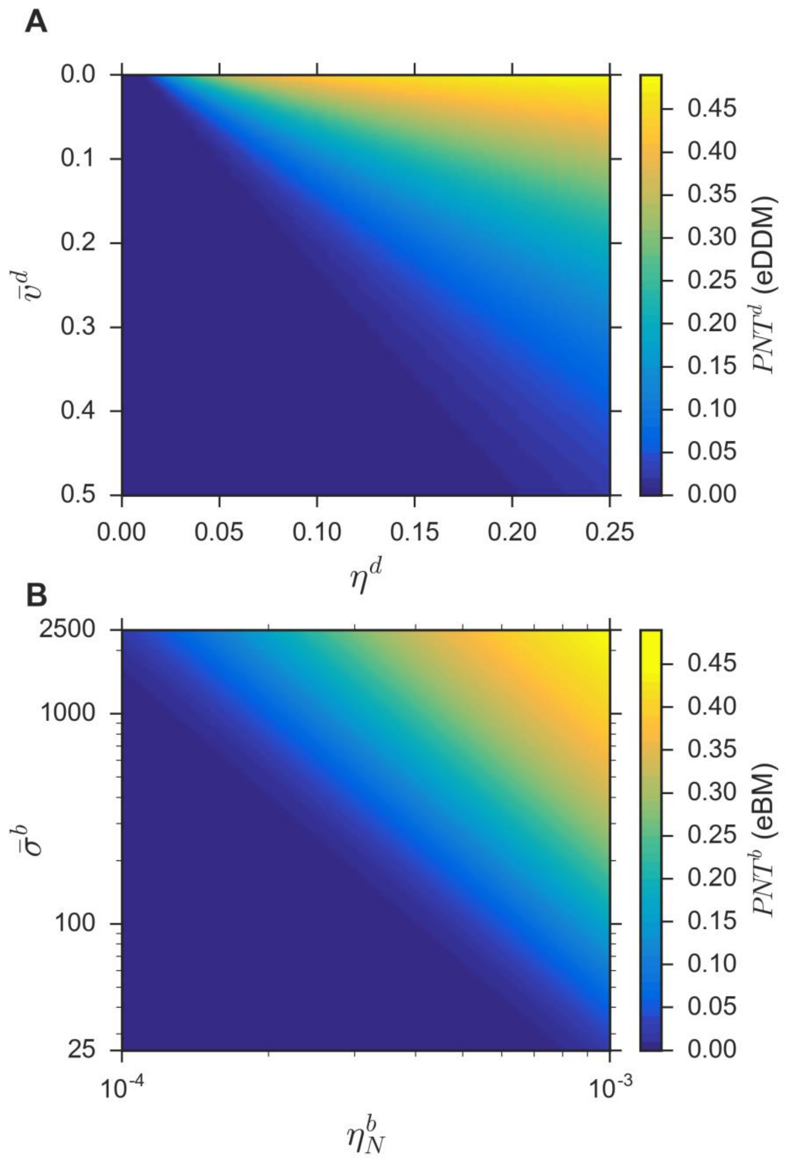

Supplementary Figure 1. Approximating the proportion of negative drift rates ($\mathbf{PNT^d}$). **(A)** The $\mathbf{PNT^d}$ in the eDDM (Eq. 18) as a function of mean drift rate ($\bar{\mathbf{v}}^\mathbf{d}$) and variability of drift rate ($\boldsymbol{\eta}^\mathbf{d}$). The values are largest in the shown range when $\bar{\mathbf{v}}^\mathbf{d}$ is close to 0 and $\boldsymbol{\eta}^\mathbf{d}$ is close to 0.25. As the values of $\bar{\mathbf{v}}^\mathbf{d}$ increase and values of $\boldsymbol{\eta}^\mathbf{d}$ decrease the $\mathbf{PNT^d}$ becomes smaller. **(B)** Log-log plot of approximated $\mathbf{PNT^b}$ in the eBM (Eq. 19) as a function of mean noise level ($\bar{\boldsymbol{\sigma}}^\mathbf{b}$) and variability of the noise level ($\boldsymbol{\eta}^\mathbf{b}_\mathbf{N}$). As $\bar{\boldsymbol{\sigma}}^\mathbf{b}$ decreases (increasing $\bar{\mathbf{v}}^\mathbf{d}$) and $\boldsymbol{\eta}^\mathbf{b}_\mathbf{N}$ decrease, the values of $\mathbf{PNT^b}$ decrease.

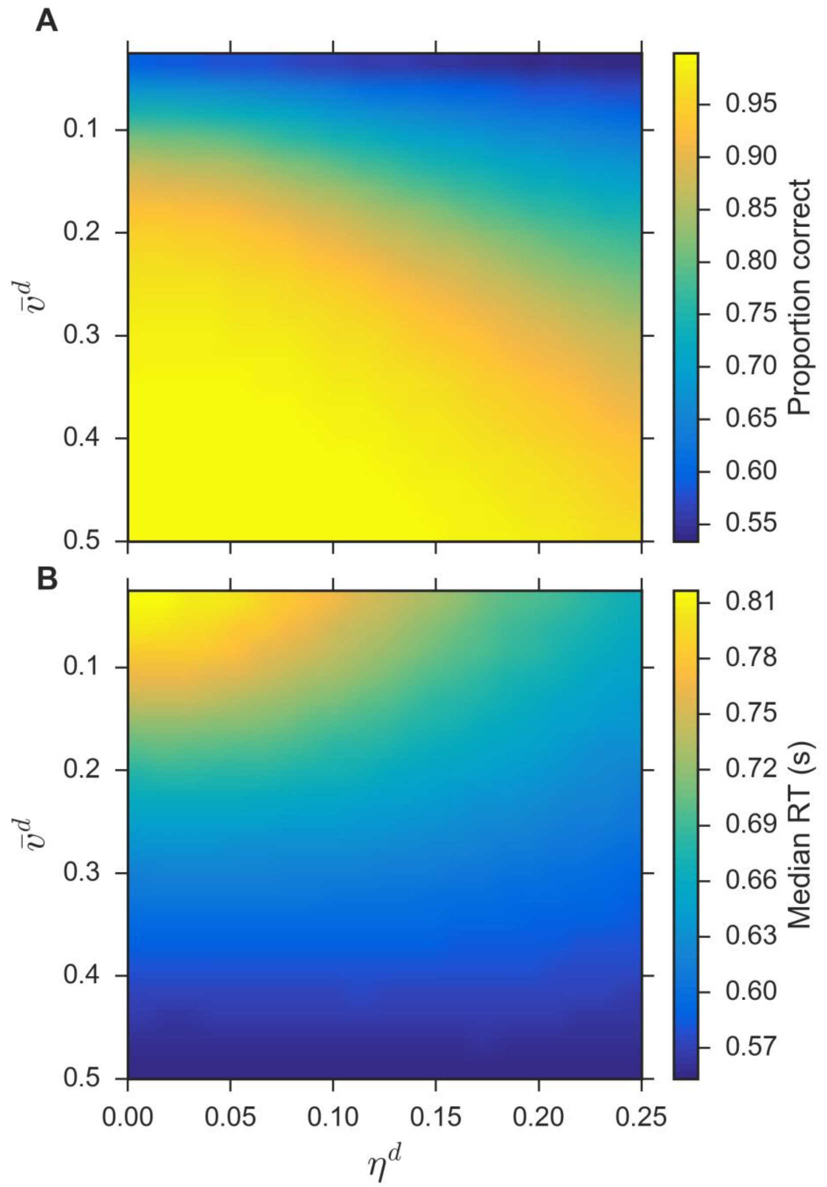

Supplementary Figure 2. Modelling the effect of task difficulty with the eDDM. **(A)** the proportion correct i.e. accuracy and **(B)** median RT as a function of mean drift rate ($\bar{\mathbf{v}}^{\mathbf{d}}$) and variability of drift rate ($\boldsymbol{\eta}^{\mathbf{d}}$) parameters. As typically found with the eDDM, as $\bar{\mathbf{v}}^{\mathbf{d}}$ decreases with a given a fixed η^{d} value, the proportion correct decreases and RTs become slower. With higher $\boldsymbol{\eta}^{\mathbf{d}} > \mathbf{0.15}$, The proportion correct decreases, and for $\bar{\mathbf{v}}^{\mathbf{d}} < \mathbf{0.2}$, the median RT decreases (similar effects have been observed in the eBM simulations; see Figure 5.A,B). For generating the plots, we varied the two parameters $\bar{\mathbf{v}}^{\mathbf{d}}$ and $\boldsymbol{\eta}^{\mathbf{d}}$ in the range ($\mathbf{0} < \bar{\mathbf{v}}^{\mathbf{d}} < \mathbf{0.5},\ \mathbf{0} < \boldsymbol{\eta}^{\mathbf{d}} < \mathbf{0.25}$) while fixing the remaining parameters ($\mathbf{B}^{\mathbf{d}} = \mathbf{0.06},\ \Delta\mathbf{t} = \mathbf{0.05},\ \mathbf{s}^{\mathbf{d}} = \mathbf{0.1},\ \bar{\mathbf{z}}^{\mathbf{d}} = \mathbf{0},\ \mathbf{s}_{\mathbf{Z}}^{\mathbf{d}} = \mathbf{0}\ ,\ \mathbf{T}_{\mathbf{nd}}^{\mathbf{d}} = \mathbf{0.4},\ \mathbf{s}_{\mathbf{t}}^{\mathbf{d}} = \mathbf{0.2}$). We simulated a dataset with 100,000 decisions for each parameter-set. Responses with RT bigger than 5000 ms were marked as timed-out trials.

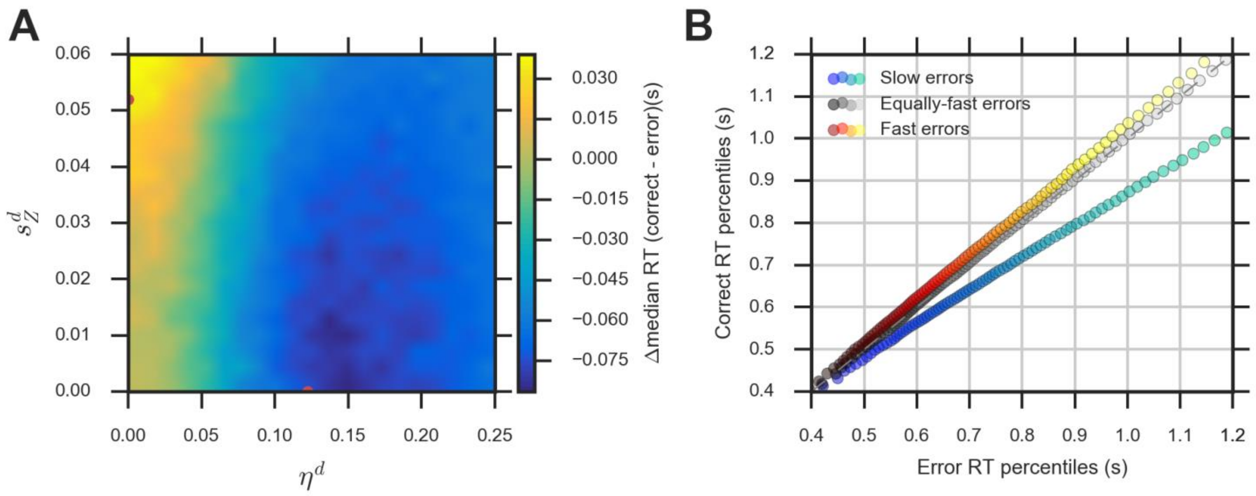

Supplementary Figure 3. The eDDM accounts of slow and fast errors. **(A)** The median RT difference between correct and error responses. The two red dots indicate the example parameter values chosen for demonstrating the slow and fast errors effect in **(B)** Quantile-quantile plot which shows how the eDDM captures either slow or fast errors. The three dotted lines depict correct and error RT percentiles (up to 100 dots per line, each dot corresponds to one of the percentiles from 1st to 100th) from three datasets generated by varying the values of $\boldsymbol{\eta^d}$ and $\mathbf{s_Z^d}$. For all three lines, a few percentiles representing very long RTs (>1200 ms, above 95th percentile) are not shown in the plot. The grey dotted line represents zero variability in drift and bias: correct and error RTs are equal. The red-yellow dotted line above the dashed diagonal line shows the effect of fast errors (using $\boldsymbol{\eta^d} = \mathbf{0}$, $\mathbf{s_Z^d} = \mathbf{0.052}$) where the majority of error RT percentiles are faster than the ones for correct response. The blue-green dotted line below the diagonal line shows the effect of slow errors (using $\boldsymbol{\eta^d} = \mathbf{0.122}$, $\mathbf{s_Z^d} = \mathbf{0}$) . For generating **(A)**, we varied the two parameters $\boldsymbol{\eta^d}$ and $\mathbf{s_Z^d}$ in the range ($\mathbf{0} < \boldsymbol{\eta^d} < \mathbf{0.25}$, $\mathbf{0} < \mathbf{s_Z^d} < \mathbf{0.06}$) and for generating both plots we the remaining parameters ($\mathbf{B^d} = \mathbf{0.06}$, $\boldsymbol{\Delta}\mathbf{t} = \mathbf{0.05}$, $\mathbf{s^d} = \mathbf{0.1}$,$\bar{\mathbf{v}}^{\mathbf{d}} = \mathbf{0.133}$, $\bar{\mathbf{z}}^{\mathbf{d}} = \mathbf{0}$, $\mathbf{T_{nd}^d} = \mathbf{0.4}$, $\mathbf{s_t^d} = \mathbf{0.2}$). We simulated a dataset with 100,000 decisions for each parameter-set. Responses with RT bigger than 5000 ms were marked as timed-out trials.

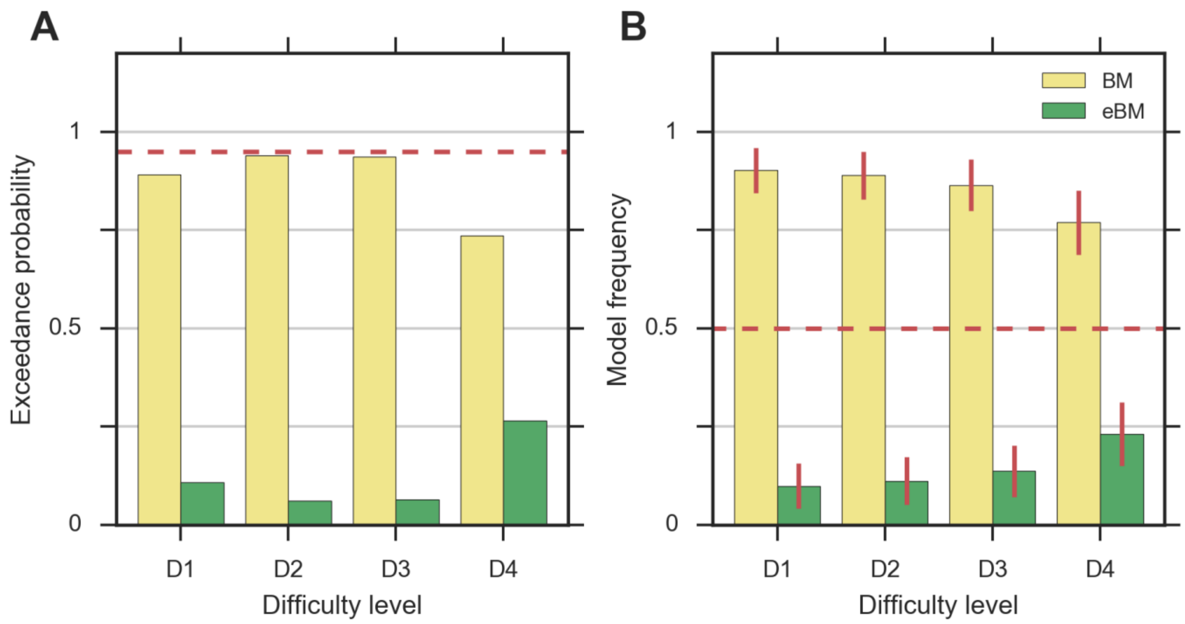

Supplementary Figure 4. Results of random-effects Bayesian model comparison between BM and eBM across four difficulty conditions, **(A)** Protected exceedance probability and **(B)** Model frequency. Protected exceedance probability is the estimated probability that the given family of models wins model comparison in the population of participants. The model frequency refers to the probability that a randomly selected participant behaves according to the considered model. As expected, there is considerable evidence for the BM with exceedance probabilities nearly reaching 95% in conditions D1-D3 and a slightly lower value in the easiest condition (D4). The model frequency is above 75% in all conditions. The small rise in exceedance probabiliy and model frequency for the eBM in the easiest condition hints at the effect present in **Figure 7**, but without exact input there is clearly no evidence for benefits of parameter variability in explaining the responses of the participants. For **(A)** the red dashed line represents the threshold usually used to indicate very strong evidence for a model (0.95). For **(B)** the red dashed line indicates chance level and error bars indicate the posterior standard deviation of the estimated model frequencies.
